# Sox8 and Sox9 regulate differentiation and nuclear positioning of retinal Müller glia

**DOI:** 10.1242/dev.205508

**Published:** 2026-10-13

**Authors:** Nicole Pannullo, Debarpita Datta, Clayton P. Santiago, Jared A. Tangeman, Lizhi Jiang, Leighton H. Duncan, Taqdees Gohar, Megan Gimmen, Seth Blackshaw

**Affiliations:** ^1^ Solomon H. Snyder Department of Neuroscience, University of Pittsburgh, Pittsburgh, PA 15219, USA; ^2^ Department of Ophthalmology, University of Pittsburgh, Pittsburgh, PA 15219, USA; ^3^ Department of Ophthalmology, Johns Hopkins University School of Medicine, Baltimore, MD 21287, USA; ^4^ Department of Neurology, Johns Hopkins University School of Medicine, Baltimore, MD 21287, USA; ^5^ Kavli Neuroscience Discovery Institute, Johns Hopkins University School of Medicine, Baltimore, MD 21287, USA; ^6^ Institute for Cell Engineering, Johns Hopkins University School of Medicine, Baltimore, MD 21287, USA

**Keywords:** Retina, Temporal patterning, Cell migration, Cell adhesion, Müller glia, Oligodendrocyte

## Abstract

Temporal patterning of retinal progenitor cells governs the sequential generation of retinal cell types, with gliogenesis occurring late in development. Sox8 and Sox9, members of the SoxE transcription factor family, are highly expressed in late-stage retinal progenitor cells and mature Müller glia, yet their functional roles remain incompletely defined. Here, we have employed gain- and loss-of-function approaches, single-cell multiomic profiling, and injury models to investigate Sox8 and Sox9 function. Overexpression of human *SOX8* and *SOX9* in early-stage mouse retinal progenitor cells both suppressed generation of early-born cells, consistent with a role in late-stage temporal identity, and induced ectopic generation of oligodendrocyte lineage-like cells. Conversely, conditional deletion of *Sox8* and/or *Sox9* in late-stage progenitors did not impair Müller glia specification, but caused radial displacement of glial nuclei into the outer retina and reduced expression of multiple markers of mature Müller glia. Loss of *Sox8* and/or *Sox9* in mature Müller glia modestly increased proliferation post-injury without inducing neurogenic competence. These findings suggest that *Sox8* and *Sox9* are dispensable for gliogenesis and repression of neurogenic competence, but are essential for proper laminar positioning and full terminal differentiation of retinal Müller glia.

## Teaser

**Summary:** Gain- and loss-of-function approaches, single-cell multiomic profiling, and injury models reveal that Sox8 and Sox9 are dispensable for Müller glia specification but essential for their laminar positioning and terminal differentiation.

## INTRODUCTION

Cell fate specification in the developing nervous system is largely directed by temporal patterning and involves a process known as developmental competence. This competence is characterized by dynamic changes in gene expression and regulation throughout developmental neurogenesis that mediate the ability of progenitors to generate individual cell types. Single-cell multiomic analyses of multiple regions in the developing vertebrate central nervous system (CNS) have identified gene regulatory networks that correlate directly with early and late stages of developmental competence (Lyu et al., 2021; Sagner et al., 2021; Kim et al., 2025; La Manno et al., 2021; Telley et al., 2019). These networks in turn include several transcription factors that show conserved patterns of temporal regulation across multiple CNS regions. Several of the transcription factors identified in the gene regulatory network active in late-stage progenitors were first implicated in controlling the generation of glia, which are invariably among the last-born cell types (Miller and Gauthier, 2007; Frisén, 2016). These include members of the nuclear factor I (NFI) and SoxE gene families (*Sox8*, *Sox9* and *Sox10*). Overexpression of both *Nfia* and *Sox9* induce astrogliogenesis in the brain, while loss of function compromises gliogenesis (Tchieu et al., 2019; Deneen et al., 2006; Takouda et al., 2021; Klum et al., 2018; Stolt et al., 2003). In the retina, overexpression of multiple NFI family members simultaneously suppresses the generation of early-born cell types while promoting formation of late-born cell types and mitotic exit, whereas loss of function has the opposite effect (Lyu et al., 2021; Clark et al., 2019). Moreover, NFI factors and Sox9 frequently bind to common target sites controlling expression of genes that promote both late-stage temporal identity and gliogenesis (Kang et al., 2012). This has raised the possibility that NFI and SoxE factors may form an essential core component of the gene regulatory network that controls these processes (Zhang et al., 2023).

Since the major cell types of the retina are generated exclusively through dynamic changes in temporal patterning (Santos-França et al., 2023; Cepko, 2014), it provides an ideal model for investigating this topic mechanistically. Müller glia (MG), the principal glial cells of the retina, emerge late in retinal development from retinal progenitor cells (RPCs) during a shift from neurogenesis to gliogenesis that coincides with increased expression of both NFI and SoxE factors (Lyu et al., 2021; Clark et al., 2019). The SoxE gene family includes *Sox8*, *Sox9* and *Sox10*. While *Sox10* is not detectably expressed in neuroretina, *Sox8* and *Sox9* are robustly expressed in late-stage RPCs and their expression is maintained in mature MG (Clark et al., 2019; Lu et al., 2020). RPC-specific loss of function of *Sox9* has been reported to eliminate expression of mature MG markers without disrupting retinal integrity (Poché et al., 2008), while shRNA-mediated knockdown of either *Sox8* or *Sox9* in late-stage RPCs was reported to promote generation of rod photoreceptors at the expense of MG (Muto et al., 2009). This suggests that Sox8 and Sox9 may both be required to both promote retinal gliogenesis and suppress neurogenesis in late-stage RPCs.

This in turn raises the question as to whether sustained expression of *Sox8* and/or *Sox9* in mature MG is required to repress neurogenic competence. Both loss of function of NFI factors and disruption of Notch signaling in mature MG induce neurogenic competence and lead to the generation of late-born inner retinal neurons; in the case of *Nfia*, *Nfib* and *Nfix*, their loss also induces limited MG proliferation (Le et al., 2024; Hoang et al., 2020). In both cases, downregulation of *Sox8* and *Sox9* expression is also observed. This led us to hypothesize that these SoxE factors are themselves downstream targets of NFI and Notch signaling, and that their sustained expression may inhibit efficient MG reprogramming. We suspected that Sox8 and Sox9 may not only function as markers of mature MG identity, but may also act directly to enforce glial quiescence and suppress latent neurogenic programs.

In this study, we investigated the role of Sox8 and Sox9 in both developing and mature MG using both overexpression in early-stage RPCs and conditional loss of function in both late-stage RPCs and mature MG. While overexpression of *SOX8* and/or *SOX9* inhibited formation of early-born retinal ganglion cells, as expected, we did not observe any changes in retinal cell fate following *Sox8* and/or *Sox9* loss of function in late-stage RPCs. Instead, we observed displacement of MG nuclei into the outer nuclear layer (ONL), along with a modest reduction in a subset of mature MG markers. Selective disruption of *Sox8* and/or *Sox9* in mature MG likewise did not induce glial-derived neurogenesis, in either unstimulated or injury-induced conditions, but did result in a modest increase in proliferation immediately following excitotoxic injury. Single-cell multiomic analysis of mutant cells revealed widespread changes in expression of genes controlling cell adhesion and migration in addition to mature glial markers, along with reduced accessibility of chromatin regions targeted by Sox8 and Sox9. We conclude that while Sox8 and Sox9 influence gene expression and nuclear positioning in MG, they are ultimately dispensable for retinal gliogenesis.

## RESULTS

### *Sox8* and *Sox9* are expressed in late-stage RPCs and mature Müller glia

We first sought to confirm previous reports of the cellular expression pattern of *Sox8* and *Sox9* in the developing mouse retina. Analyzing scRNA-Seq data collected across the full time course of mouse retinal development (Fig. S1A) revealed that both *Sox8* and *Sox9* show substantially higher expression in late-stage RPCs relative to early-stage RPCs, with this being particularly so for *Sox8*. Sustained expression is observed in differentiated MG at postnatal day (P)14, but expression is not detected in retinal neurons at this age (Fig. S1B). Cellular expression levels of both *Sox8* and *Sox9* peak from E18-P2, in line with the transition from early- to late-stage identity, which occurs around E17 in mice (Lyu et al., 2021; Clark et al., 2019) (Fig. S1C). In adult retina, *Sox8* and *Sox9* expression is maintained in MG, with limited levels also observed in retinal astrocytes but not in subtypes of retinal neurons (Fig. S1D).

### Overexpression of *SOX8* and/or *SOX9* in early-stage RPCs suppresses generation of early-born cell types

To determine whether overexpression of *SOX8* and/or *SOX9* was sufficient to regulate temporal patterning in early-stage RPCs, we electroporated early-stage developing retinal explants with either control pCAGIG or plasmids overexpressing *SOX8* and *SOX9*, either individually or in combination (Fig. 1A). The explants were electroporated at embryonic day (E) 14 and cultured until E19 (P0), with an EdU pulse delivered at E16 (Fig. 1B). We then used immunohistochemistry to analyze changes in cell composition and proliferation in GFP-positive electroporated cells. Overexpression of *SOX8*, and to a lesser extent *SOX9*, led to a reduction in the percentage of GFP- and Rbpms-positive retinal ganglion cells (RGCs) (Fig. 1C,D). The effect was not significantly enhanced by overexpression of both *SOX8* and *SOX9* in combination (Fig. 1C,D). In contrast, we observed increased numbers of GFP and Otx2-positive photoreceptor precursor cells, with this effect being statistically significant when *SOX8* and *SOX9* were overexpressed together (Fig. 1E,F). This matches the shift in temporal identity observed following overexpression of *Nfia*, *Nfib* and *Nfix* in early-stage RPCs (Lyu et al., 2021). We also observed a statistically significant decrease in the proportion of GFP-positive amacrine cells (ACs) with overexpression of *SOX8* compared to the control (Fig. 1G,H). Interestingly, there was a similar but slightly weaker effect with *SOX9* or combined overexpression (Fig. 1G,H). Fewer GFP and Sox2-positive RPCs were observed with *SOX8* and combined *SOX8* and *SOX9* overexpression, with statistical significance only achieved with *SOX8* overexpression (Fig. S2A,B). A relative increase in GFP- and Mki67-positive cells was observed for all experimental conditions, although this effect was also only reached statistical significance following *SOX8* overexpression (Fig. S2C,D). In contrast, a statistically significant increase in EdU incorporation in GFP-positive cells was observed following overexpression of *SOX8* and *SOX9* in combination, while the increase in EdU incorporation observed following *SOX8* or *SOX9* overexpression did not reach statistical significance (Fig. S2E,F). Overall, these results may reflect transient stimulation of proliferation in RPCs followed by cell cycle exit and depletion of primary RPCs, again consistent with a shift towards later-stage temporal identity. Disordered lamination, particularly of Rbpms-positive retinal ganglion cells, was also frequently observed following overexpression of *SOX8* and *SOX9*, either individually or in combination, consistent with disrupted expression of genes controlling cell adhesion and lamination in RPCs (Fig. 1C).

**Fig. 1.**
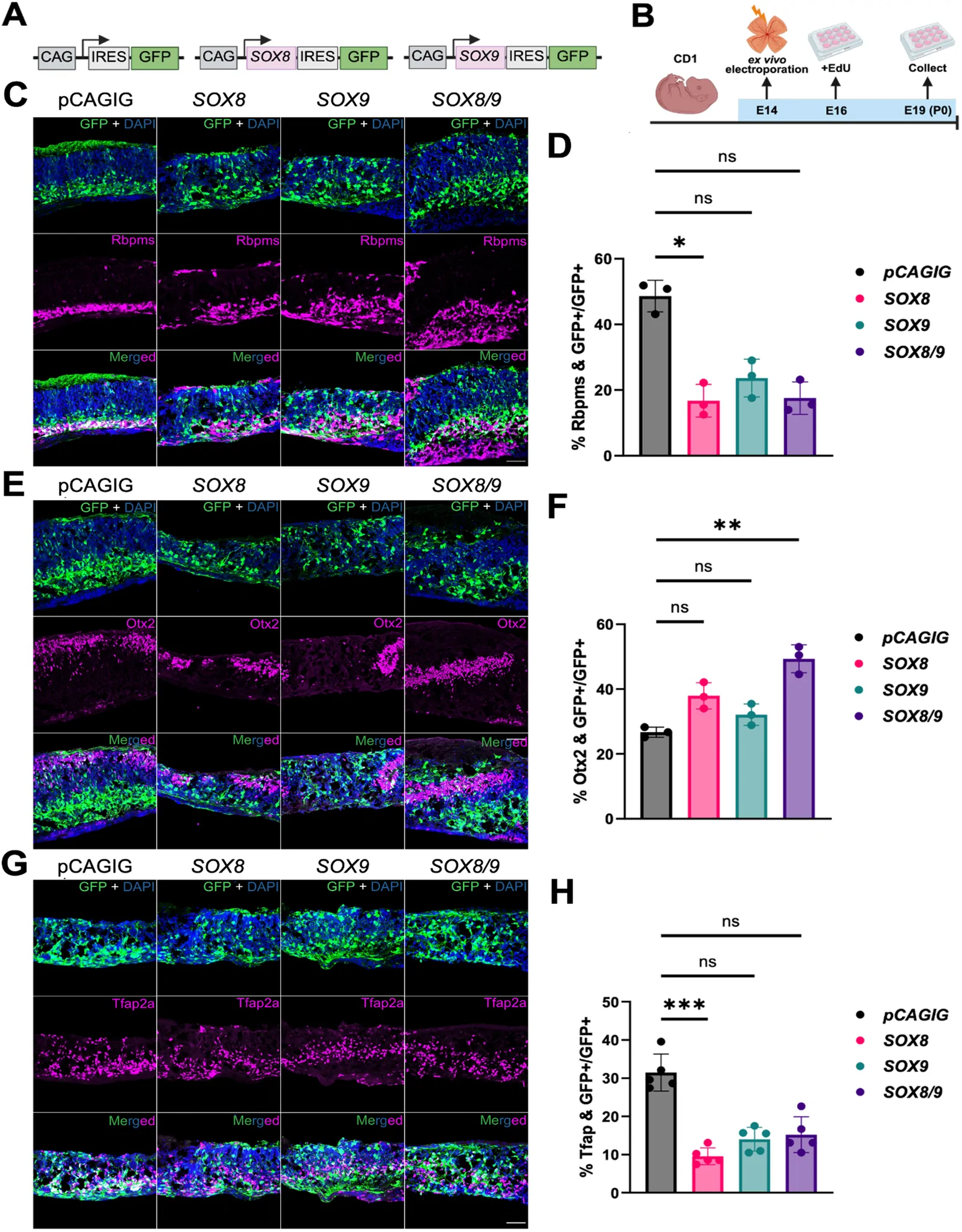
**Overexpression of human *SOX8* and *SOX9* in retinal explant cultures**. (A) Schematic of the constructs used to induce expression of green fluorescent protein (GFP), *SOX8* and *SOX9*. (B) Schematic of the experimental workflow. P0 retinal explants were collected following *ex vivo* electroporation at E14 with control (pCAGIG), *SOX8*, *SOX9*, or pooled *SOX8* and *SOX9* constructs. (C,E,G) Representative immunostaining for GFP and retinal cell type markers. (C) Rbpms (retinal ganglion cells), (E) Otx2 (photoreceptors) and (G) Tfap2a (amacrine cells) in electroporated retinal explants collected at P0. Scale bars: 50 μm. DAPI, 4′,6-diamidino-2-phenylindole. (D,F,H) Quantification of mean percentage±s.d. of GFP-positive electroporated cells that co-label with (D) Rbpms, (F) Otx2 or (H) Tfap2a for each condition. Significance was determined using Kruskal–Wallis with Dunn's multiple comparisons: *P<0.0332, **P<0.0021, ***P<0.0002; ns, not statistically significant. Each data point was calculated from an individual retina.

To analyze changes in gene expression resulting from overexpression of *SOX8* and *SOX9*, we performed scRNA-Seq analysis on GFP-positive cells that were FACS-isolated from P0 retinal explants electroporated at E14 with either the control pCAGIG or both *SOX8* and *SOX9* overexpression plasmids (Fig. S3A). All retinal cell types appropriate for the developmental stage were identified (Fig. S3B). Most strikingly (Pozniak et al., 2010), *SOX8* and *SOX9* overexpression resulted in the formation of a *Sox10*/*Nkx2-2*/*Olig1*-positive cell cluster that expressed multiple oligodendrocyte-lineage (OL) genes (Fig. S3C). These included both oligodendrocyte progenitor cell (OPC)-enriched genes, such as *Cspg4*, *Pdgfra* and *Gpr17*, and myelin-related genes such as *Plp1*, *Mbp* and *Mobp*. In contrast to the increased proportion of Otx2 and GFP double-positive cells observed using immunohistochemistry, we did not observe an overall increased percentage of photoreceptor precursors and rods with *SOX8* and *SOX9* overexpression (Fig. S3D). However, *SOX8* and *SOX9* overexpression did result in a decreased percentage of RPCs and early-born cell types, including cones, RGCs and ACs (Fig. S3D). This is consistent with the overall decrease in the proportion of Sox2 and GFP, Rbpms and GFP, and Tfap and GFP double-positive cells observed with immunohistochemical analysis (Fig. 1B,C,F,G and Fig. S2A,B).

Direct comparison of gene expression profiles between control RPCs and RPCs overexpressing *SOX8* and *SOX9* identified 300 differentially expressed genes. Upregulated genes include transcription factors that are known or predicted to promote late-stage temporal identity, such as *Tox*, *Sox6*, *Nfia* and *Nfib*, along with well-established late-stage RPC markers, such as *Cd44* (Lyu et al., 2021; Clark et al., 2019) (Fig. S3E, Table S1). 22/72 (30.6%) of upregulated genes are also selectively enriched in late-stage RPCs in developing retina, while only 2/72 (2.4%) are enriched in early-stage RPCs (Fig. S3F, Table S1). In contrast, roughly similar numbers of early-stage (17/228) and late-stage (23/228) RPC-enriched genes were downregulated by *SOX8* and *SOX9* overexpression, although these included the early-stage temporal identity regulator *Foxn4* and the well-established early-stage RPC marker *Fgf15* (Liu et al., 2020; Blackshaw et al., 2004) (Fig. S3E,F, Table S1). Taken together, these indicate that Sox8 and Sox9 repress the generation of early-born cells, broadly induce genes specific to late-stage temporal identity and are capable of inducing ectopic generation of oligodendrocyte lineage cells when overexpressed in the retina.

### Loss of function of either *Sox8* or *Sox9* in late-stage RPCs leads to radial displacement of MG nuclei towards the ONL

We first attempted to generate RPC-specific conditional mutants of *Sox8* and *Sox9* by crossing to the RPC-specific *Chx10-Cre* transgenic line (Rowan and Cepko, 2004). We successfully generated *Chx10-Cre;Sox9^lox/lox^* mice and collected retinas at P14 and P120 for scRNA-Seq analysis (Fig. S4A,B). Efficient deletion of exons 2 and 3 was observed in *Sox9*-deficient retinas (Fig. S4C), and all major retinal cell types, including MG, were identified at both timepoints (Fig. S4D,E). Direct comparison of gene expression profiles between wild-type control and *Sox9*-deficient MG revealed 497 differentially expressed genes (104 upregulated and 393 downregulated) at P14 and 682 differentially expressed genes (293 upregulated and 389 downregulated) at P120 (Table S2). Small defects in Sox family (*Sox2*, *Sox5* and *Sox6*) and MG-specific (*Glul* and *Abca8a*) gene expression, along with slight induction of genes that promote neurogenesis (*Ascl1* and *Btg2*), were observed at P14, with an even more subtle effect observed at P120 (Fig. S4F-H). Gene set enrichment analysis of differentially expressed genes in P14 Sox9-deficient MG showed slight activation of relevant gene sets, including neuron differentiation and generation of neurons (Fig. S4I). However, immunohistochemical analysis revealed no obvious defects in retinal neurogenesis, glial specification or glial morphology.

We were also unable to generate any homozygous *Chx10-Cre;Sox8^lox/lox^* pups, despite many rounds of breeding. We therefore instead selectively disrupted *Sox8* and *Sox9* in late-stage RPCs using both the *RaxCreER* knock-in and the *GlastCreER* transgenic line, both of which have been previously shown to work effectively for this purpose (de Melo et al., 2012; Pak et al., 2014), in combination with the Cre-inducible *R26-CAG-lsl-Sun1-GFP* line, which labels the nuclear envelope (Mo et al., 2015). Cre recombination was induced in pups via intramuscular injection of tamoxifen from P3-5, and retinas were collected for immunohistochemical analysis at P60 (Fig. 2A-C). Both treatments efficiently induced loss of Sox8 immunoreactivity (Fig. 2D), and also led to mosaic loss of Sox9 immunoreactivity (Fig. 2E). Loss of function of either gene leads to displacement of MG nuclei towards the outer nuclear layer, as detected by Sox2 immunoreactivity, with a stronger phenotype observed for *Sox8* mutants (Fig. 2F,G), although this may reflect the more efficient Cre-dependent loss of function of this gene. All displaced cells expressed Sox2, indicating that glial identity is preserved, in contrast to previous reports (Poché et al., 2008).

**Fig. 2.**
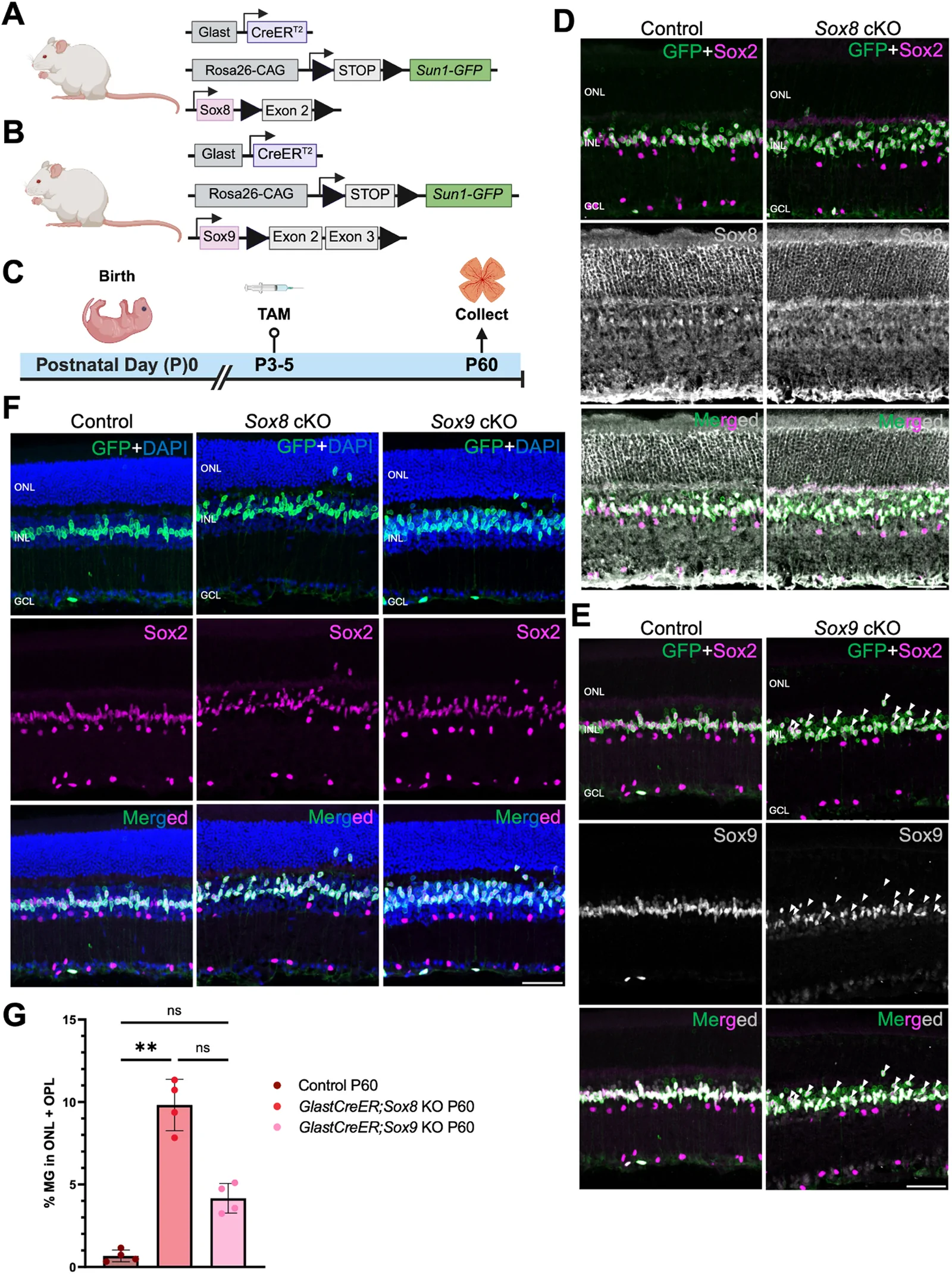
**Selective loss of function of either *Sox8* or *Sox9* in late-stage retinal progenitor cells leads to radial displacement of Müller glia nuclei to the outer nuclear layer/outer plexiform layer.** (A,B) Schematics of the transgenic constructs used to induce deletion of *Sox8* or *Sox9* specifically in late-stage retinal progenitor cells (RPCs)/Müller glia (MG). Cre-mediated removal of exon 2 of *Sox8* or *Sox9* leads to premature stop codon formation and disrupts the expression of the Sox8 or Sox9 protein. (C) Schematic of the experimental workflow. (D) Representative immunostaining for green fluorescent protein (GFP), Sox8 and Sox2 in control and *Sox8-*deleted retinas collected at postnatal day (P) 60. (E) Representative immunostaining for GFP, Sox9 and Sox2 in control and *Sox9-*deleted retinas collected at P60. White arrowheads indicate co-labeled GFP and Sox2-positive cells that are Sox9 negative. (F) Representative images showing DAPI, GFP and Sox2 in control and either *Sox8-* or *Sox9-*deleted retinas collected at P60. The control images are the same as those in E. ONL, outer nuclear layer; INL, inner nuclear layer; GCL, ganglion cell layer. DAPI, 4′,6-diamidino-2-phenylindole. (G) Quantification of mean percentage±s.d. of GFP-positive MG displaced to either the outer nuclear layer (ONL) or outer plexiform layer (OPL). Significance was determined using Kruskal–Wallis with Dunn's multiple comparisons: **P<0.0021; ns, not statistically significant. Each data point was calculated from an individual retina. Scale bars: 50 μm.

### Combined loss of function of both *Sox8* and *Sox9* in late-stage RPCs leads to increased numbers of radially displaced MG

We next tested the effects of combined loss of function of *Sox8* and *Sox9* in late-stage RPCs, again using both *RaxCreER* and *GlastCreER*, and the *R26-CAG-lsl-Sun1GFP* line (Fig. 3A,E), and administering tamoxifen from P3-5. The retinas were then collected at P16 and P60 or P90 for immunohistochemical analysis (Fig. 3B,F). In both cases, we observed substantial levels of MG displacement at P16, with 15% of GFP-positive MG nuclei radially displaced in *RaxCreER*;*Sox8^lox/lox^;Sox9^lox/lox^*;*R26-CAG-lsl-Sun1GFP* (Fig. 3C,D) and 25% of *GlastCreER*;*Sox8^lox/lox^;Sox9^lox/lox^* ;*R26-CAG-lsl-Sun1GFP* (Fig. 3G-I) displaced towards or in the outer nuclear layer (ONL). At P90, this increased to 31% in *RaxCreER*;*Sox8^lox/lox^;Sox9^lox/lox^*;*R26-CAG-lsl-Sun1GFP* mice (Fig. 3D), but no significant age-dependent increase in displacement is seen with *GlastCreER*;*Sox8^lox/lox^;Sox9^lox/lox^* ;*R26-CAG-lsl-Sun1GFP* mice at P60 (Fig. 3I).

**Fig. 3.**
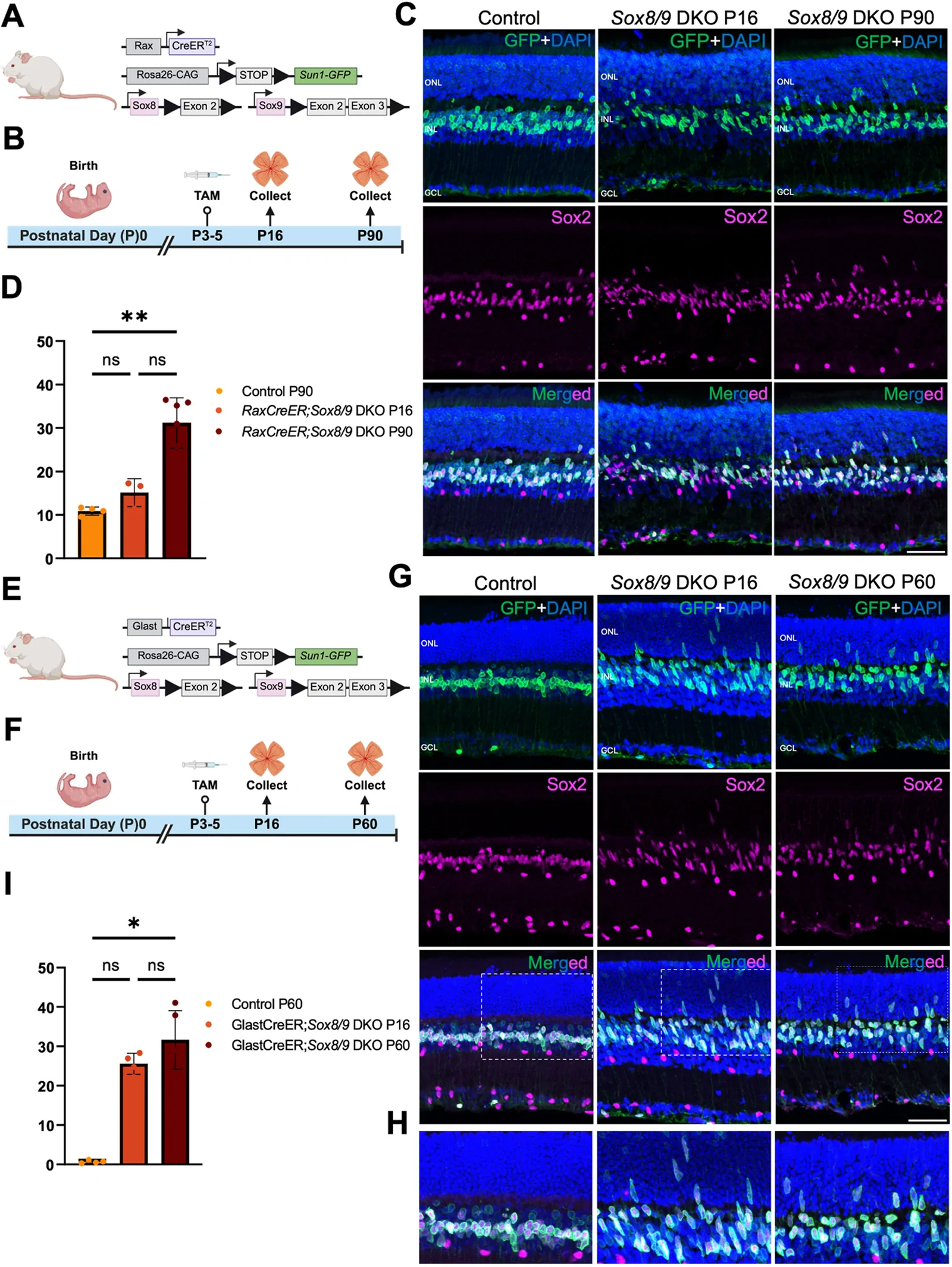
**Selective loss of function of *Sox8* and *Sox9* during late-stage retinal development enhances Müller glia displacement to the outer nuclear layer/outer plexiform layer.** (A,E) Schematics of the transgenic constructs used to induce deletion of *Sox8* and *Sox9* specifically in late-stage retinal progenitor cells (RPCs) and Müller glia (MG). Cre-mediated removal of exon 2 of *Sox8* and *Sox9* leads to premature stop codon formation and disrupts the expression of the Sox8 and Sox9 proteins. (B,F) Schematics of the experimental workflow. (C,G,H) Representative immunostaining for GFP and Sox2 in control and *Sox8-* and *Sox9*-deleted retinas collected at P16 and either P90 (C) or P60 (G,H). Scale bars: 50 μm. (D,I) Quantification of mean percentage±s.d. of GFP-positive MG displaced to either the outer nuclear layer (ONL) or outer plexiform layer (OPL). Significance was determined using Kruskal–Wallis with Dunn's multiple comparisons: *P<0.0332, **P<0.0021; ns, not statistically significant. Each data point was calculated from an individual retina.

At all timepoints, we also observe that most displaced MG nuclei show elongated morphology, in contrast to GFP-positive cells that remained in the INL (Fig. 3C,G,H). We unexpectedly observed that 10% of MG in *RaxCreER*;*R26-CAG-lsl-Sun1GFP* control mice also show nuclear displacement at P90 (Fig. 3C,D). Since the *RaxCreER* knock-in line disrupts expression at the endogenous *Rax* locus, this suggests that this may represent a previously unreported effect of *Rax* heterozygosity, which has been observed in adult hypothalamus (Miranda-Angulo et al., 2013). For this reason, along with the more efficient overall disruption of *Sox9* expression, we therefore used the *GlastCreER*;*Sox8^lox/lox^;Sox9^lox/lox^* for the remainder of these studies.

### Combined loss of function of both *Sox8* and *Sox9* in late-stage RPCs does not grossly compromise glial-specific gene expression or retinal lamination

We next used immunostaining to determine whether there were any obvious disruptions in MG-specific gene expression or morphology in P60 *GlastCreER*;*Sox8^lox/lox^;Sox9^lox/lox^* ;*R26-CAG-lsl-Sun1GFP* mice. We observed a reduction in glutamine synthetase staining in the inner nuclear layer, although signal was preserved in both apical and basal MG processes (Fig. S5A). In contrast, we did not observe any obvious changes in Aqp4 distribution (Fig. S5B), nor did we observe any induction of GFAP (Fig. S5C) or loss of Hes1 expression (Fig. S5D). Radial displacement of MG nuclei did not affect expression of markers of the inner limiting membrane such as Lama1 (Fig. S5E) or ZO-1 (Fig. S5F). Finally, in contrast to *Nfia-*, *Nfib-*, *Nfix-* and/or *Rbpj*-deficient MG (Le et al., 2024; Hoang et al., 2020), we did not observe any cells displaced towards or in the ONL that expressed the high levels of Pax6 that are characteristic of amacrine and ganglion cells (Fig. S5G) or the bipolar cell and photoreceptor marker Otx2 (Fig. S5H).

### Single-cell multiome and CUT&RUN analysis identify Sox8 and Sox9-regulated genes

To comprehensively analyze changes in gene expression and chromatin accessibility resulting from loss of function of *Sox8* and *Sox9*, we performed combined snRNA- and ATAC-Seq multiome analysis on GFP-positive cells that were FACS isolated from P17 *GlastCreER*;*Sox8^lox/lox^;Sox9^lox/lox^* ;*R26-CAG-lsl-Sun1GFP* mice, in parallel with age-matched *GlastCreER*;*R26-CAG-lsl-Sun1GFP* controls (Fig. 4A). While GFP labeled both late-born rod photoreceptors and bipolar cells, as well as MG, we focused exclusively on MG for the purpose of this analysis. UMAP analysis of MG from control and *Sox8* and *Sox9* mutants revealed extensive overlap in gene expression profiles, but substantial divergence of a subset of mutant MG (Fig. 4B).

**Fig. 4.**
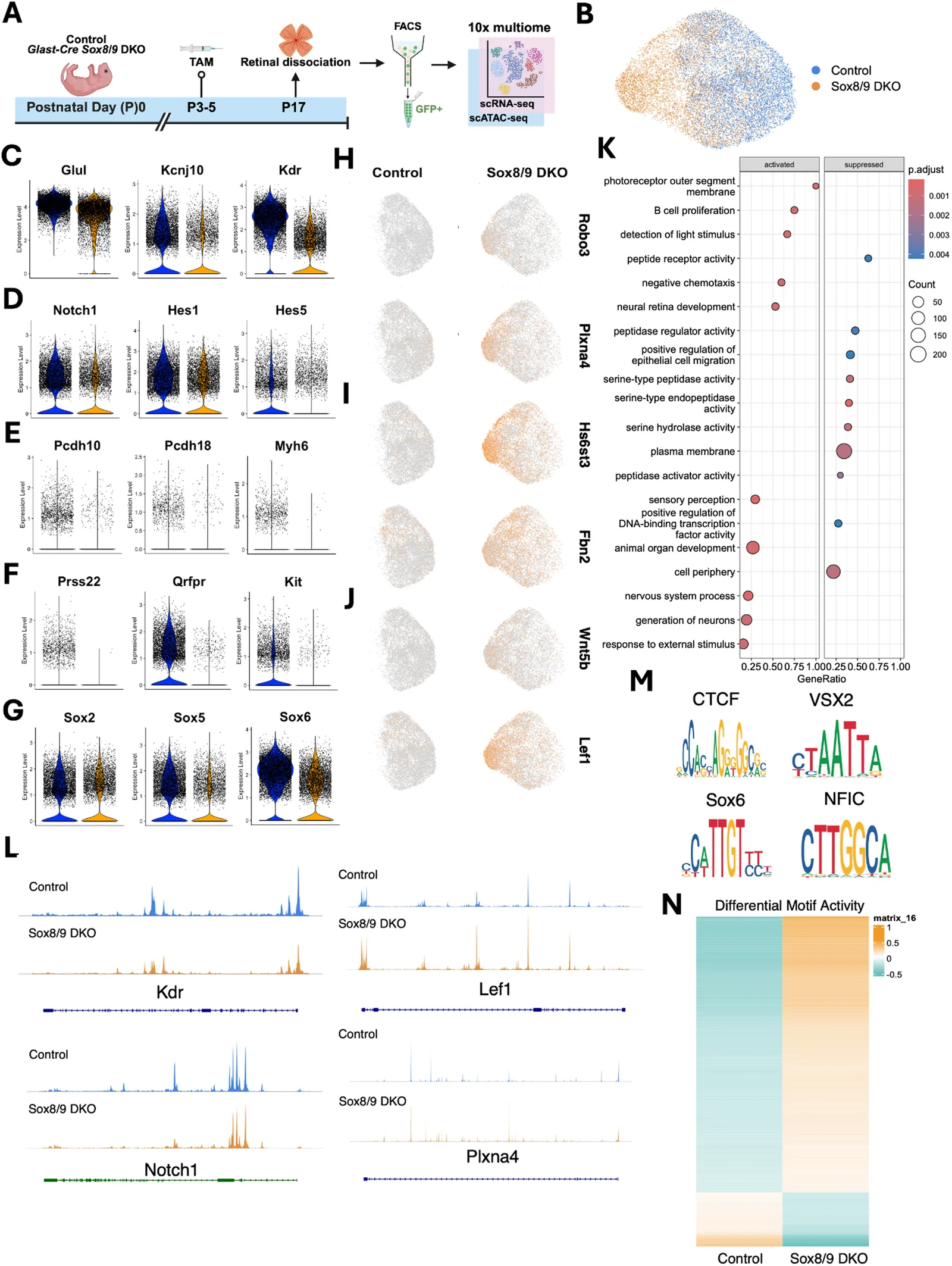
**Multiome analysis of control and *Sox8* and *Sox9* double-knockout Müller glia.** (A) Schematic of the multiome experimental pipeline. (B) UMAP plot of multiome datasets showing the clustering of control and *Sox8-* and *Sox9*-deficient Müller glia (MG). (C-G) Violin plots showing decreased expression of (C) MG markers (*Glul*, *Kcnj10* and *Kdr*), (D) Notch signaling genes (*Notch1*, *Hes1* and *Hes5*), (E) regulators of cell adhesion (*Pcdh10* and *Pcdh18*) and myosin-dependent motility (*Myh6*), (F) cell migration genes (*Prss22*, *Qrfpr* and *Kit*), and (G) Sox genes (*Sox2*, *Sox5* and *Sox6*). (H-J) Feature plots showing increased expression of (H) regulators of cell migration and/or synaptogenesis (*Robo3* and *Plxna4*), (I) extracellular matrix genes (*Hs6st3* and *Fbn2*), and (J) Wnt signaling genes (*Wnt5b* and *Lef1*). (K) Gene set enrichment analysis of significant differentially expressed genes. (L) Coverage plots for a subset of differentially expressed genes from C and D. (M) Depleted motifs in *Sox8-* and *Sox9*-deficient MG. (N) Heatmap showing differential motif activity between conditions.

Direct comparison of gene expression profiles between control and *Sox8* and *Sox9* mutant MG identified 2336 differentially expressed genes, with 460 upregulated and 1876 downregulated (Table S3). We observe reduced expression of multiple MG-specific genes in the mutants, with significant reductions of *Glul* (glutamine synthetase), the potassium channel *Kcnj10* and the VEGF receptor *Kdr* (Fig. 4C). Notch pathway genes (*Notch1*, *Hes1* and *Hes5*) were also moderately downregulated (Fig. 4D). The most strongly downregulated genes were enriched for regulators of cell adhesion (*Pcdh10*, *Pcdh18* and *Alcam*), tumor cell migration (*Prss22*, *Kit* and *Qrfpr*) or myosin-dependent motility (*Myh6* and *Myof*) (Fig. 4E,F). Several other Sox family members, including *Sox5* and *Sox6*, were also strongly downregulated, with *Sox2* also showing a modest reduction (Fig. 4G). Strongly upregulated genes included regulators of neuronal cell migration and synaptogenesis (*Robo3* and *Plxna4*), extracellular matrix (*Hs6st3* and *Fbn2*) or Wnt signaling (*Lef1* and *Wnt5b*) (Fig. 4H-J). Notably, although the progressive radial displacement of MG nuclei that we observe following *Sox8* and *Sox9* loss of function (Fig. 3D,I) is reminiscent of proliferation-induced interkinetic nuclear migration (Goldman, 2014), we did not observe any detectable increase in cell cycle gene expression, suggesting that this displacement did not reflect active cell proliferation. Gene set enrichment analysis of differentially expressed genes identified several functional categories that matched these observations, including negative chemotaxis in upregulated genes and positive regulation of epithelial cell migration in downregulated genes (Fig. 4K). These are consistent with Sox8 and Sox9 playing a general role in regulating adhesion and radial positioning of MG.

We next analyzed the scATAC-Seq data to identify differentially accessible regions associated with these genes. 3924 differentially accessible regions were identified, with 1109 showing increased accessibility and 2815 having reduced accessibility (Table S3). We observed a widespread and consistent reduction in accessibility in regulatory regions associated with downregulated genes (Fig. 4L). SOX family motifs, including Sox8 and Sox9 consensus sites, were the most strongly enriched motifs in regions that showed reduced accessibility in mutants, as expected (Fig. 4M,N). RORB and FOS/JUN motifs were also enriched in these regions. Motifs showing increased accessibility were enriched for CTCF and multiple Lhx family members, which likely correspond to Lhx2, which promotes gliogenesis and represses reactive gliosis (Zibetti et al., 2019; de Melo et al., 2016b, 2012).

To determine whether any major changes in MG-specific gene expression were observed in older animals, we also performed scRNA-Seq analysis of P90 *RaxCreER*;*Sox8^lox/lox^;Sox9^lox/lox^* ;*R26-CAG-lsl-Sun1GFP* and *RaxCreER*;*R26-CAG-lsl-Sun1GFP* littermate controls (Fig. S6A). All major retinal cell types, including MG, were identified in similar proportions at this timepoint (Fig. S6B-D). Direct comparison of MG-specific gene expression profiles revealed some similar patterns of differential gene expression. This includes modest downregulation of several mature MG markers (*Glul* and *Kcnj10*), Notch pathway components (*Notch1* and *Hes1*), cell migration genes (*Qrfpr*) and myosin-dependent motility genes (*Myof*), along with upregulation of genes associated with cell migration and/or synaptogenesis (*Plxna4*), and Wnt signaling (*Lef1*) (Fig. S6E). Sox family factors (*Sox2*, *Sox3*, *Sox4*, *Sox5*, *Sox6*, *Sox8*, *Sox9* and *Sox11*) were also generally moderately downregulated (Fig. S6F). 1675 differentially expressed genes were identified, with 1094 upregulated and 581 downregulated (Table S4, Fig. S6G). However, gene set enrichment analysis of differentially expressed genes did not identify functional categories that matched these observations, which may correlate with the milder phenotype that we observed using the *RaxCreER* transgenic line (Fig. S6H).

We next performed CUT&RUN analysis to identify direct targets of Sox8 and Sox9 at both P2 and P17 in wild-type mouse retinas (Fig. 5A). The data were integrated with both previously published scATAC-Seq data from P2 retina (Lyu et al., 2021) and P17 retinal scATAC-Seq data obtained from multiomic analysis of both control and Sox8 and Sox9 double-knockout MG (Fig. 4A). We detected 5616 Sox8 and/or Sox9 peaks in P2 retinas, with 437 of these peaks overlapping with differentially accessible regions (DARs) detected in P2 RPCs (Fig. 5B, Table S5). Gene set enrichment analysis of these overlapping peaks revealed enrichment of multiple developmental processes, including regulation of neurogenesis, eye development, axon guidance, and cell fate commitment (Fig. 5C). At both ages, we observed that both Sox8 and Sox9 mainly bind to DARs located within 1 kb of transcription start sites (TSS) (Fig. 5D,E). The great majority of Sox8 and Sox9 peaks associated with DARs present in P2 RPCs overlapped with H3K27ac, and in P17 MG with both H3K27ac and H3K4me3, indicating that they represent active promoter or enhancer elements (Fig. 5F,G; Fig. S7A,B). Substantial overlap was observed between peaks detected at P2 and P17 using two independent monoclonal antibodies against Sox8 (Fig. S7C,D), and between Sox8 and Sox9 peaks at both ages (Fig. S7E). Loss of function of *Sox8* and *Sox9* led to a dramatic reduction in accessible chromatin at these sites, indicating that Sox8 and Sox9 are essential for maintaining chromatin accessibility at their target sites (Fig. 5F).

**Fig. 5.**
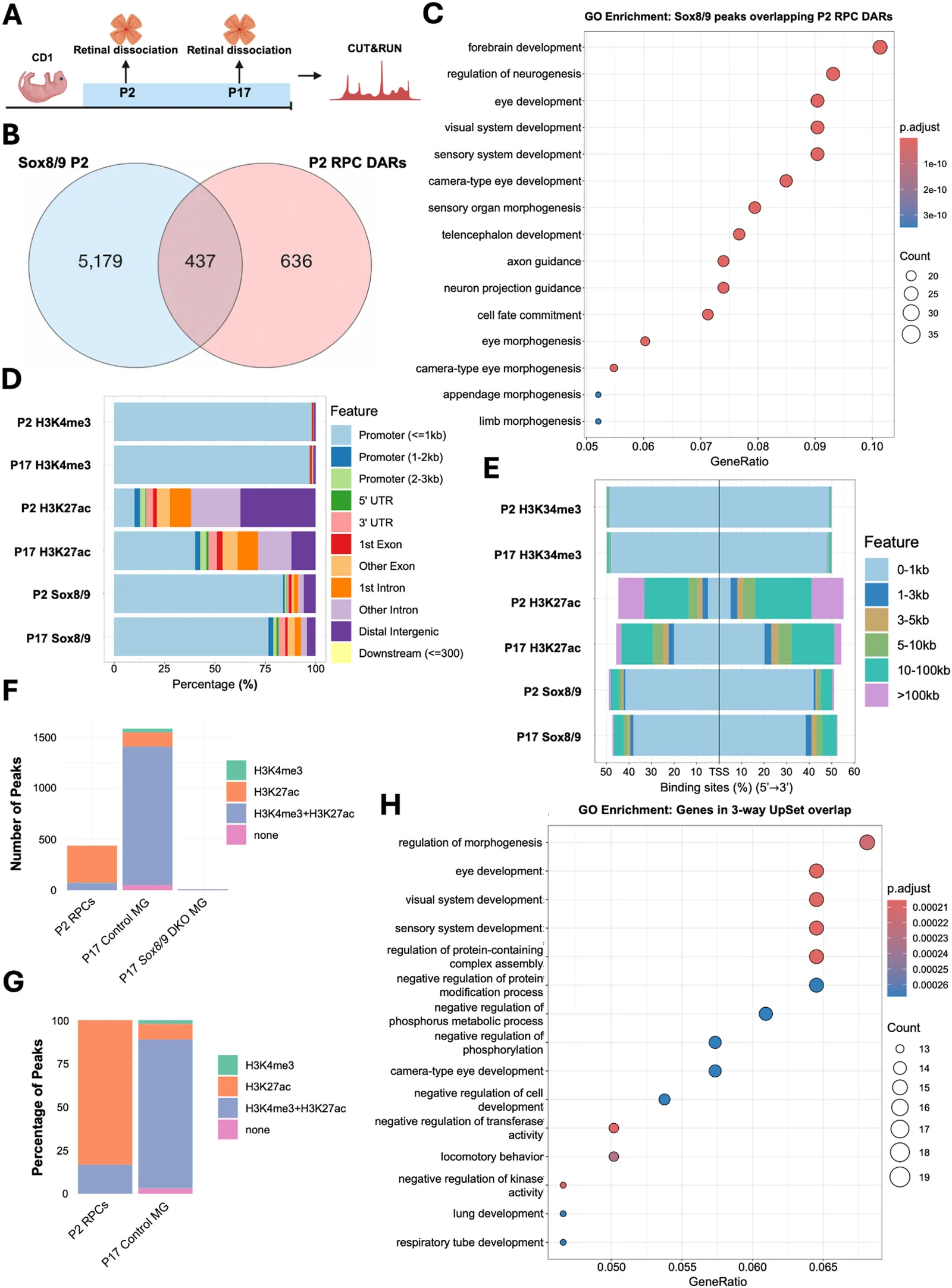
**CUT&RUN analysis of Sox8 and Sox9 in P2 and P17 mouse retina.** (A) Schematic of the CUT&RUN experimental pipeline. (B) Overlap of P2 Sox8 and Sox9 peaks with P2 retinal progenitor cell (RPC) differentially accessible regions (DARs). (C) GO enrichment of P2 Sox8 and Sox9 peaks, and P2 RPC DARs. (D) Distribution of Sox8- and Sox9-binding loci in distinct gene regions at P2 and P17 compared to H3K27ac and H3K4me3 controls. (E) Distribution of Sox8- and Sox9-binding loci relative to transcription start site (TSS) at P2 and P17 compared to H3K27ac and H3K4me3 controls. (F) Number of Sox8 and Sox9 CUT&RUN peaks that overlap with enriched regions or DARs in either P2 RPCs, P17 control Müller glia (MG) or P17 *Sox8* and *Sox9* double-knockout MG. (G) Distribution of Sox8 and Sox9 CUT&RUN peaks that overlap with enriched regions or DARs in either P2 RPCs or P17 control MG. (H) Gene ontology enrichment of 3-way overlapping *Sox8* and *Sox9* double-knockout differentially expressed genes, DARs, and P17 Sox8 and Sox9 peaks.

Gene set enrichment analysis of 3-way overlapping peaks showed enrichment of cellular processes relevant to the phenotype observed in *Sox8* and *Sox9*-deficient MG, including regulation of morphogenesis and eye development, and negative regulation of cell development and locomotory behavior (Fig. 5H). We also saw that both Sox8 and Sox9 are preferentially associated with accessible chromatin regions that are enriched for both Sox family motifs and the RPC- and MG-enriched homeodomain factor Lhx2, which ATAC-Seq analysis has revealed is crucial for sustaining accessibility of regulatory regions targeted by transcription factors that promote late-stage temporal identity (Lyu et al., 2021; Zibetti et al., 2019) (Table S5). Motifs for KLF family transcription factors, multiple members of which are enriched in late-stage RPCs (Clark et al., 2019), are also observed, particularly at P2. Sox8 and Sox9 peaks overlap with motifs for multiple factors – including CTCF, RORB and FOS/JUN – that also show reduced accessibility in scATAC-Seq analysis of P17 *Sox8* and *Sox9* mutant MG (Fig. 4M,N). We observe that *Sox5*, *Sox8* and *Sox9* are all directly targeted by Sox8 and/or Sox9 at P2, and Sox8- and Sox9-binding sites are also enriched for other classes of transcription factors, notably Sp1 and ZBTB family members, the expression of which is differentially regulated in *Sox8* and *Sox9* mutants. Furthermore, at both P2 and P17, we see that Sox8 and/or Sox9 directly target regulatory sequences associated with Notch pathway genes (*Notch1*, *Hes1* and *Hes5*), as well as MG-specific genes (*Kcnj10*, *Kdr* and *Fxyd3*) and genes regulating cell migration and adhesion (*Cdh2*, *Ddr1*, *Pcdh10* and *Kit*) that are differentially expressed in *Sox8* and *Sox9* double knockout MG (Tables S3 and S5).

### Loss of function of *Sox8* and/or *Sox9* in mature MG induces limited levels of proliferation but does not induce neurogenic competence

Finally, to investigate whether loss of function of *Sox8* and *Sox9* either individually or in combination was sufficient to induce either proliferation or neurogenesis in mature MG, we administered tamoxifen-supplemented chow to P21 mice for 3 weeks (Fig. 6A). To create injury conditions that are permissive for glial-derived neurogenesis (Le et al., 2024; Jorstad et al., 2017), at 1 week following resumption of normal chow, we induced excitotoxic damage by intravitreal injection as described previously (Hoang et al., 2020) and pulsed with EdU for 3 consecutive days immediately following this. We then harvested retinas at 4 weeks post-injury to investigate injury-induced proliferation of GFP-positive MG. As in our model of *Sox8* and *Sox9* loss of function in late-stage RPCs, we observed efficient *Sox8* deletion following the TAM diet regimen, while *Sox9* deletion was mosaic (Fig. 6B). *Sox8*- and *Sox9*-deficient MG nuclei are radially displaced towards the outer plexiform layer (OPL) (Fig. 6B), although, in contrast to loss of function at P3-5 (Fig. 3), no GFP-positive nuclei are observed within the ONL in the absence of injury. While we observe minimal levels of EdU incorporation in control animals, we observe a significant increase in EdU induction in both *Sox8*, and *Sox8* and *Sox9* double mutants (Fig. 6C,D). Both EdU-positive and EdU-negative *Sox8*- and *Sox9*-deficient GFP-positive cells are immunoreactive for Sox2 (Fig. 6E). No GFP-positive cells express Ki67, indicating that EdU incorporation occurred transiently immediately following injury (Fig. 6F). The presence of small numbers of GFP-positive nuclei in the ONL of NMDA-treated animals is likewise consistent with proliferation-induced interkinetic nuclear migration (Goldman, 2014). In addition, we observe that GFP-positive MG also largely continue to express the Notch pathway gene Hes1 (Fig. S8A), do not express the neurogenic bHLH factor Ascl1 (Fig. S8B), and do not show any co-expression of GFP with selective markers of late-born retinal neurons (Fig. S8C,D), indicating that *Sox8*- and/or *Sox9*-deficient MG lack neurogenic competence even after excitotoxic injury.

**Fig. 6.**
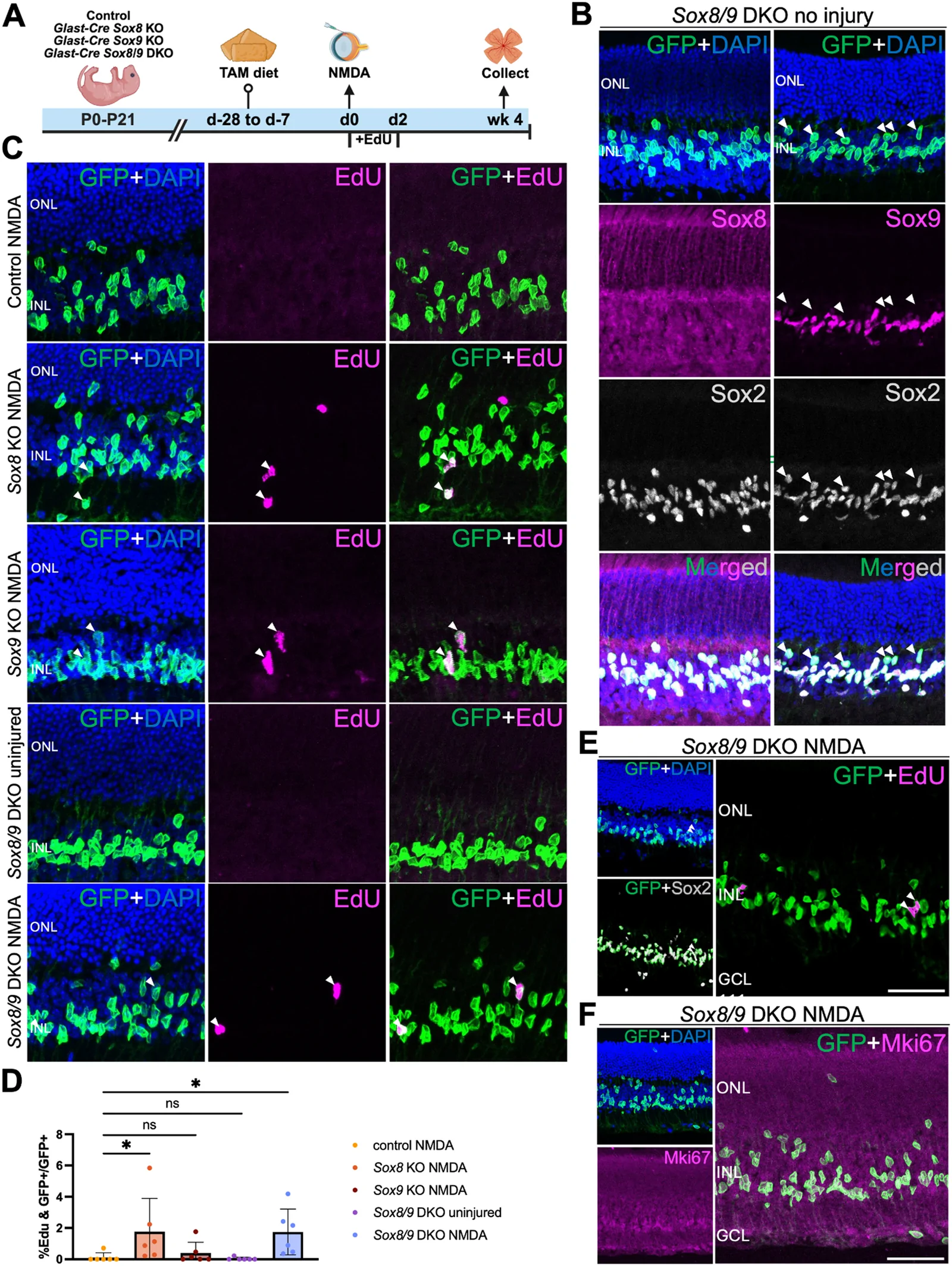
**Selective loss of function of *Sox8* and *Sox9* in mature Müller glia stimulates proliferation with retinal injury**. (A) Schematic of the experimental workflow. (B) Representative immunostaining for GFP and Sox8 or Sox9 in uninjured *Sox8-* and *Sox9*-deleted retinas 5 weeks after finishing the tamoxifen (TAM) diet. White arrowheads indicate co-labeled GFP- and Sox2-positive cells that are Sox9 negative, which are radially displaced towards the outer plexiform layer (OPL) relative to Sox9-positive Müller glia (MG) in the same section. (C) Representative immunostaining for GFP and EdU in control, *Sox8-*, *Sox9-*, and *Sox8-* and *Sox9*-deleted retinas collected 4 weeks following retinal injury. White arrowheads indicate co-labeled GFP-positive and EdU-positive cells. (D) Quantification of mean percentage±s.d. of EdU-positive MG. Significance was determined using Kruskal–Wallis with Dunn's multiple comparisons: *P<0.0332; ns, not statistically significant. Each data point was calculated from an individual retina. (E,F) Representative immunostaining for GFP, and Sox2 (E) or Mki67 (F) in *Sox8-* and *Sox9*-deleted retinas collected 4 weeks following retinal injury. Scale bars: 50 μm.

## DISCUSSION

Transcription factors that control late-stage temporal identity in neural progenitors play a central role in the specification of late-born neurons and glia, and in repressing proliferative and neurogenic competence in mature glia (Clark et al., 2019). In light of both their expression pattern and their predicted function in gene regulatory networks reconstructed from single-cell multiomic analysis (Lyu et al., 2021), SoxE factors are prime candidates for playing a central role in this process. In this study, we systematically reassess the roles of the SoxE factors Sox8 and Sox9 across retinal development and in mature MG using temporally controlled genetic perturbations and single-cell genomic approaches. We find that overexpression of *SOX8* and/or *SOX9* in early retinal progenitors is sufficient to both broadly induce expression of late-stage RPC genes and inhibit generation of early-born retinal cell types, particularly retinal ganglion cells, consistent with their predicted role in promoting late-stage temporal identity. Genetic loss of function of *Sox8* and *Sox9*, either individually or in combination, does not alter retinal cell fate specification, refining earlier models that proposed an essential role for SoxE factors in MG specification (Muto et al., 2009; Poché et al., 2008). Likewise, we do not observe induction of neurogenesis in *Sox8*- and *Sox9*-deficient MG, even under injury paradigms previously shown to robustly induce neurogenesis following loss of function of *Nfia/b/x* or Notch signaling (Le et al., 2024; Hoang et al., 2020). Instead, we observe a reproducible but restricted phenotype characterized by radial displacement of a subset of MG, modest changes in mature glial gene expression, and a limited injury-induced proliferative response that does not progress to neurogenesis.

Our observation that *SOX8* and *SOX9* overexpression suppresses generation of early-born cell types is consistent with these genes being able to modulate temporal patterning at this age, but does not imply that they can also do so when strong determinants of temporal identity such as *Nfia*, *Nfib* and *Nfix* are expressed. Indeed, previous studies have failed to observe any increase in retinal gliogenesis following postnatal overexpression of either *Sox8* or *Sox9* (de Melo et al., 2016a). Our analyses of *Sox8* and *Sox9* mutants are also seemingly in contradiction with previous reports of an essential role for Sox8 and Sox9 in promoting retinal gliogenesis (Muto et al., 2009; Poché et al., 2008). Using multiple temporally and cell type-restricted genetic strategies, we do not observe the loss of MG previously reported following *Sox9* disruption, suggesting that earlier findings may reflect differences in timing or targeting strategies. Another study that used shRNA to reduce *Sox8* and *Sox9* expression reported an increase in photoreceptor generation and a corresponding reduction in MG generation (Muto et al., 2009). This, however, was conducted from E17.5 onwards, in contrast to our own work in which *Sox8* and/or *Sox9* were disrupted at later stages; this, in addition to potential off-target effects of the constructs used, may reconcile our observations. Finally, a more recent study reported rapid rod photoreceptor degeneration following global conditional disruption of *Sox9* in adult mice (Hurtado et al., 2025). This in turn may reflect a disruption of the visual cycle due to loss of *Sox9* function in the retinal pigment epithelium, where it plays an essential role in regulating expression of these genes (Masuda et al., 2014).

The most prominent effect of *Sox8* and *Sox9* disruption that we observed was the radial displacement of the nuclei of a subset of MG into the outer nuclear layer, along with the adoption of an elongated nuclear morphology in these displaced cells. While Sox9 promotes migration of non-CNS cell types – most notably tumor cells (Yang et al., 2023; Wang et al., 2021; Rockich et al., 2013; Larsimont et al., 2015) – neither it nor Sox8 have been linked to the control of lamination or radial migration in the CNS. This is reminiscent of the phenotype observed following postnatal deletion of *Sox2* (Bachleda et al., 2016), although the severity is substantially greater following *Sox8* and *Sox9* deletion. This radial displacement resembles the interkinetic nuclear migration seen following injury-induced proliferation in zebrafish (Goldman, 2014), and excitotoxic injury does induce limited proliferation in adult MG lacking *Sox8* and *Sox9*. However, scRNA- and snRNA-Seq detect no cell cycle gene expression in *Sox8-* and/or *Sox9*-deficient cells at any age that we profiled, indicating that displaced cells are not proliferating. Although displacement may partly reflect abortive cell cycle re-entry in immature MG, the progressive increase in displaced nuclei from P16 to P90 in *RaxCreER*;*Sox8^lox/lox^;Sox9^lox/lox^* animals (Fig. 3D) argues that this is not the primary cause of the observed phenotype.

ScRNA-Seq analysis shows altered expression of genes encoding cell adhesion, extracellular matrix formation, cell migration and myosin-based motility. Loss of function of direct Sox8 and Sox9 target genes, such as *Kit*, *Dpysl3* and *Rnd2* have been linked to defects in cell migration in the developing CNS (Guijarro et al., 2013; Tanaka et al., 2012; Nakamura et al., 2006), and loss of function of cell-adhesion molecules such as *Pcdh10* and *Pcdh18* regulate both cell adhesion and migration (Zhen et al., 2023; Aamar and Dawid, 2008). These data support a model in which Sox8 and Sox9 function within a distinct gene regulatory module that, in combination with Sox2, Sox5 and Sox6, stabilizes MG morphology and nuclear positioning, rather than specifying fate or neurogenic competence. Future work dissecting how Sox8 and Sox9 interact with other components of late-stage glial regulatory networks will further clarify how these transcriptional regulatory networks stabilize glial identity and morphology.

Gene regulatory networks controlling temporal patterning and glial maturation are highly redundant, and our findings indicate that Sox8 and Sox9 act in a subsidiary or dependent capacity relative to core regulators such as NFI factors and Notch signaling. In contrast to perturbations of NFI factors or Notch signaling, which are both necessary and sufficient to induce robust MG reprogramming (Clark et al., 2019; Lyu et al., 2021; Le et al., 2024; Hoang et al., 2020), loss of *Sox8* and *Sox9* produces a qualitatively distinct and more constrained phenotype.

This implies that Sox8 and Sox9 have a subsidiary and dependent function in controlling both processes, which is supported by scRNA-Seq data showing reduced expression of both genes in *Nfia-*, *Nfib-*, *Nfix-* and *Rbpj*-deficient MG (Le et al., 2025; Hoang et al., 2020). Sox8 and Sox9 in turn appear to control expression of other transcription factors that are part of this late-stage RPC and glial-specific network, such as *Sox5* and *Sox6*, which in turn inhibit proliferation and modulate gliogenesis in other CNS regions (Martinez-Morales et al., 2010; Stolt et al., 2006; Lefebvre, 2010; Scheel et al., 2005). A more-complete understanding of the hierarchical organization of the transcription factor networks that maintain glial quiescence and identity will help guide ongoing efforts aimed at efficiently and safely inducing glial reprogramming for the treatment of neurodegenerative diseases and diseases that cause blindness (Blackshaw and Cayouette, 2025; Langhe and Pearson, 2020; Goldman, 2014).

Finally, our observation that combined expression of *SOX8* and *SOX9* induced ectopic formation of OL-like cells is unexpected. The mouse retina lacks oligodendrocytes entirely; although they occur in rabbit and chick retinas, these are derived from ventral forebrain-derived OPCs that migrate into the retina through the optic nerve (Morcos and Chan-Ling, 1997; Ono et al., 2017). Although overexpression of either factor accelerates differentiation of committed immature oligodendrocytes (Pozniak et al., 2010), and *Sox9* overexpression promotes formation of both OL and astrocytes in ventral spinal cord (Vogel et al., 2020), neither factor is sufficient to induce OL in the dorsal neural tube or in other regions not normally competent to generate them. This contrasts with the closely related OL-specific SoxE family member *Sox10*, which can confer at least partial competence for OL generation that is substantially enhanced by co-expression of *Olig2* (Liu et al., 2007; Pozniak et al., 2010). We observe that *SOX8* and *SOX9* overexpression not only robustly induces *Sox10* and *Olig1/2*, but also other OL-specific transcription factors such as *Nkx2-2* and *Myrf* (Fig. S3C, Table S1). However, while Sox8 and Sox9 directly regulate *Olig2* (Fig. S5), they do not regulate the other OL-specific transcription factors, and the mechanism by which they confer competence for retinal OL induction remains unclear and merits further study.

### Limitations of the study

Some caveats bear on the interpretation of these findings. First, because the *Chx10-Cre;Sox8^lox/lox^* mutant mice were unexpectedly embryonic lethal, we could not assess deletion of either *Sox8* alone or *Sox8* and *Sox9* in early-stage RPCs. Had *Sox8* – alone or in combination with *Sox9* – been essential for initiating late-stage RPC identity, our approach would have missed it. This could instead be addressed using alternative transgenic lines active in early-stage RPCs, such as *Rax-Cre* and *Six3-Cre* (Furuta et al., 2000; Swindell et al., 2006), although these are not RPC-specific like *Chx10-Cre*. Second, the shifts in retinal cell-type composition that are observed following overexpression of *SOX8* and *SOX9* using immunohistochemical markers, most notably the increased fraction of Otx2-positive cells, are not evident in the time-matched scRNA-Seq data. This may reflect the use of only two scRNA-Seq biological replicates or the unexpected post-transcriptional effects of *SOX8* and *SOX9* overexpression, and will require further study to resolve. Finally, although our single-cell RNA-Seq analysis of *Sox8-* and *Sox9-*deficient MG has identified several candidate regulators of MG nuclear position, these remain to be tested functionally.

## MATERIALS AND METHODS

### Mice

Maintenance and experimental procedures performed on mice were in accordance with the protocol approved by the Institutional Animal Care and Use Committee (IACUC) at the Johns Hopkins School of Medicine under protocol number MO22M22. Mice were raised and housed in a climate-controlled pathogen-free facility on a 14-h/10-h light/dark cycle. *RaxCreER;Sun1-GFP* was used in this study and were generated by crossing the *RaxCreER* line developed by our group and the *Sun1-GFP* line developed by and obtained from J. Nathans at Johns Hopkins University. *RaxCreER;Sox8/9^lox/lox^;Sun1-GFP* mice were generated by crossing *RaxCreER;Sun1GFP* mice with conditional *Sox8^lox.lox^* (Cambridge-Soochow University Genome Resource Center, EPD0618_3_E09) and *Sox9^lox/lox^* (The Jackson Laboratory, RRID:IMSR_JAX:013106) mice. *GlastCreER;Sun1-GFP* mice were also used in this study and were generated by crossing the *GlastCreER* and *Sun1-GFP* lines developed by J. Nathans at Johns Hopkins University and were obtained from his group. *GlastCreER; Sox8^lox/lox^;Sun1-GFP*, *GlastCreER;Sox9^lox/lox^;Sun1-GFP* and *GlastCreER;Sox8/9^lox/lox^;Sun1-GFP* mice were generated by crossing Cre-negative *Sox8/9^lox/lox^;Sun1-GFP* mice with *GlastCreER;Sun1-GFP* mice. *Chx10Cre* (Rowan and Cepko, 2004) mice were crossed with *Sox9^lox/lox^* mice to generate *Chx10Cre;Sox9^lox/lox^* mice. Timed pregnant CD1 mice were ordered from Charles River Laboratories for the explant plasmid overexpression analysis.

### Tamoxifen injection

To induce Cre recombination, animals at postnatal day (P)3 were intramuscularly injected with tamoxifen (TAM) (Sigma-Aldrich, H6278-50 mg) in corn oil (Sigma-Aldrich, C8267-500ML) at 0.5 mg per dose for 3 consecutive days.

### Tamoxifen diet

To induce Cre recombination, animals at ∼3 weeks of age were fed a TAM diet (Envigo, TD.130856) for 3 consecutive weeks.

### NMDA treatment

One week after finishing the TAM diet, adult mice were anesthetized with isoflurane inhalation. 1.5 μl of 100 mM NMDA+1 μg/ml EdU in phosphate-buffered saline (PBS) were intravitreally injected using a microsyringe with a 33-gauge blunt-ended needle.

### EdU treatment

At the same time as NMDA treatment, mice were administered EdU (Abcam, ab146186) via intraperitoneal injections [150 μl of PBS (5 mg/ml)]. Additional intraperitoneal injections were performed daily for the following 2 days, totaling three injections over 3 days.

### Retinal explant electroporation and culture

Eyes were enucleated from E14.5 CD1 embryos, and retinas were dissected in fresh, ice-cold 1× PBS, then transferred to a micro-electroporation chamber. The retinas were electroporated with plasmid solution at 30 V for 50 ms with 950 ms intervals (Matsuda and Cepko, 2008) using either a pCAGIG plasmid that expresses IRES-GFP (Matsuda and Cepko, 2004; Onishi et al., 2009), a bicistronic transcript that expresses full-length human *SOX8* or *SOX9* with IRES-GFP, or a 50:50 mixture of the *SOX8* and *SOX9* constructs (1 μg/μl). The electroporated retinas were then flattened by a series of radial cuts and mounted on 0.2 μm Nucleopore Track-Etch membranes on culture media (for 50 ml final media: 12.5 ml of HBSS, 12.5 ml of heat-inactivated horse serum, 1.45 ml of glucose solution, 25 ml of MEM, 500 μl of penicillin/streptomycin and 50 μl of L-glutamine were added) in 12-well plates and cultured until E19 (P0). At E16, retinal explants used for immunohistochemical analyses were pulsed with EdU (5 mg/ml in PBS).

### Immunohistochemistry and imaging

Collection and immunohistochemical analysis of retinas was performed as described previously (de Melo et al., 2016b). Briefly, mouse eye globes and E19 (P0) retinal explants were fixed in 4% paraformaldehyde (Electron Microscopy Sciences, 15710). Eye globes were fixed for 4 h at 4°C, while retinal explants were fixed for 2 h at 4°C. Retinas were dissected and explants were carefully detached from the membrane in 1×PBS, followed by incubation in 30% sucrose overnight at 4°C. Retinas were then embedded in frozen section medium (VWR, 95057-838), cryosectioned at 16 μm and stored at −20°C. Sections were dried for 30 min in a 37°C incubator and washed three times for 5 min with 0.1% Triton X-100 in PBS (PBST). EdU labeling was performed by using a Click-iT EdU kit (Thermo Fisher Scientific, C10340) following the manufacturer's instructions. Sections were then incubated in 10% horse serum (Thermo Fisher Scientific, 26050070), 0.2% Triton X-100 in 1×PBS (blocking buffer) for 1 h at room temperature and then incubated with primary antibodies in the blocking buffer overnight at 4°C. Primary antibodies used are listed in Table S6.

Sections were washed three times for 5 min with PBST to remove excess primary antibodies and were incubated in secondary antibodies in blocking buffer for 1 h at room temperature. Sections were then washed three times for 5 min in PBST, once in PBSm and mounted with Fluoromount-G, with DAPI (Thermo Fisher Scientific, 00-4959-52) under coverslips (VWR, 48404-453), air-dried and stored at 4°C. Fluorescent images were captured using a Zeiss LSM 700 confocal microscope. Secondary antibodies used are listed in Table S6.

### Cell quantification and statistical analysis

Sox2- and GFP-positive cells displaced to the ONL or OPL were counted and divided by the total number of Sox2- and GFP-positive cells from a single random whole section per retina. EdU- and GFP-positive cells were counted and divided by the total number of GFP-positive cells from a single random whole section per retina. Each data point in the bar graphs was calculated from an individual retina. All cell quantification data were analyzed using Kruskal–Wallis with Dunn's multiple comparisons and plotted using GraphPad Prism 10. All results are presented as means±s.d. Raw cell counts are listed in Table S7.

### Retinal cell dissociation

Retinas were dissected in fresh ice-cold PBS and retinal cells were dissociated using an optimized protocol, as previously described (Le et al., 2024). Each retinal explant scRNA-Seq sample contains the following number of retinas from animals of mixed sex: pCAGIG-1, 16 retinas; pCAGIG-2, 17 retinas; *SOX8/9*-1, 18 retinas; *SOX8/9*-2, 24 retinas. Each scRNA-Seq sample from the transgenic KO experiments contains two retinas from two animals of each sex. Each multiome sample contains a minimum of six retinas from six animals of both sexes. Each CUT&RUN sample contains six retinas from three animals of both sexes. Dissociated cells were resuspended in ice-cold Hibernate Buffer A GlutamaxBuffer containing Hibernate A (BrainBits, HALF500), B-27 supplement (Thermo Fisher Scientific, 17504044) and GlutaMAX (Thermo Fisher Scientific, 35050061).

### Single-cell RNA-Seq library preparation

scRNA-Seq was prepared on dissociated retinal cells using the 10× Genomics Chromium Single Cell 3′ Reagents Kit v3 or v4 (10× Genomics). Libraries were constructed following the manufacturer's instructions and were sequenced using Illumina NovaSeq X. Sequencing data were processed through the Cell Ranger 8.0.1 pipeline (10× Genomics) using default parameters.

### Single-cell multiome ATAC and GEX sequencing library preparation

scATAC-Seq and scRNA-Seq were prepared on FACS-isolated GFP-positive cells using the 10X Genomic Chromium Next GEM Single Cell Multiome ATAC and Gene Expression kit following the manufacturer's instructions. Nuclei were isolated using an optimized protocol previously described (Santiago et al., 2023). Briefly, cells were spun down at 500 ***g*** for 5 min and resuspended in 100 μl of ice-cold lysis buffer. The cells were then lysed by pipette-mixing four times and incubated on ice for 4 min total. Nuclei were washed with 0.5 ml of ice-cold wash buffer and spun down at 500 ***g*** for 5 min at 4°C. Nuclei pellets were resuspended in 15 μl of nuclei buffer and counted using DAPI. Resuspended nuclei (10,000 to 15,000) were used for transposition and loaded into the 10× Genomics Chromium Single Cell system. ATAC libraries were amplified with seven PCR cycles and were sequenced on Illumina NovaSeq X with ∼400 million reads per library. RNA libraries were amplified from cDNA with 14 PCR cycles and were sequenced on the Illumina NovaSeq X platform.

### Single-cell RNA-Seq data analysis

scRNA-Seq data were analyzed using the Seurat R package (Stuart et al., 2021). Briefly, gene expression data were jointly normalized by the function “NormalizeData”. The principal components analysis, dimensionality reduction, Louvain clustering and UMAP visualization were performed on the top 15 principal components. Expression of known marker genes from previous studies were used to confirm the appropriate assignment of cell types, and for identification of early and late-stage RPC-enriched genes (Lyu et al., 2021; Liu et al., 2023). Expression of significantly differentially expressed genes in RPCs and MG was analyzed using the “FindMarkers” function. Multiomic analysis of control and *Sox8/Sox9* double-knockout MG was performed using the Seurat and Signac workflow (Hao et al., 2024). Motif annotations were added using JASPAR2024 core vertebrate position frequency matrices, and transcription factor (TF) activity was quantified with chromVAR (Schep et al., 2017; Rauluseviciute et al., 2024). Differentially expressed genes (DEGs) and differential chromatin accessibility regions (DARs) between *Sox8/Sox9* double-knockout and control MG were identified using Seurat's FindMarkers with a logistic regression test for ATAC peaks using ATAC depth as a latent variable and standard Wilcoxon tests for RNA, and applying an adjusted p<0.05 threshold. DARs were annotated to nearest genes using ClosestFeature. Gene ontology enrichment of DEGs was performed using clusterProfiler (Yu et al., 2012).

### CUT&RUN library preparation and sequencing

C57BL/6J mice were euthanized and retinae were isolated in ice-cold PBS containing 1× cOmplete EDTA-free Protease Inhibitor Cocktail (Millipore Sigma, 11873580001). Two biological replicates were collected from P2 and P17 mice, each containing a pool of 6 retinae from 3 mice. Retinas were collected into microcentrifuge tubes and snap frozen by submerging in an isopentane bath on liquid nitrogen for 1 min and held at −80°C until nuclei extraction was performed. For nuclei extraction, the described protocol was followed, with minor modification (Tangeman et al., 2025). In brief, 1× cOmplete EDTA-free Protease Inhibitor Cocktail was added to all solutions, and lysis was performed by incubating tissue for 5 min in a lysis buffer containing 10 mM Tris-hydrochloride (pH 7.4) (Millipore Sigma, 93313-1L), 10 mM NaCl (Millipore Sigma, S5150-1L), 3 mM MgCl_2_ (Millipore Sigma, M1028), 0.01% Tween-20 (Bio-Rad, 1610781), 0.01% NP-40 Surfact-Amps Detergent Solution (ThermoFisher Scientific, 85124), 1% bovine serum albumin (Miltenyi Biotec, 130-091-376) and 1× cOmplete EDTA-free Protease Inhibitor Cocktail (Millipore Sigma, 11873580001), with gentle trituration throughout the incubation. Ice-cold CUT&RUN wash buffer was prepared according to manufacturer's guidance (EpiCypher, 14-1048) and was used as substitute for the final nuclei wash and resuspension. 600,000 nuclei were loaded per CUT&RUN reaction for use with the Epicypher CUT&RUN protocol v5.1 and the CUTANA ChIC/CUT&RUN Kit (EpiCypher, 14-1048). A 1.8× SPRI bead ratio was used for the final recovery of CUT&RUN DNA.

The following antibody concentrations were used in the study: 0.5 μg/reaction IgG (EpiCypher, 13-0042K), 0.5 μg/reaction H3K4me3 (EpiCypher, 13-0060K), 0.5 μg/reaction H3K27ac (EpiCypher, 13-0059), 0.5 μg/reaction SOX8 (DSHB, DA11B7), 0.5 μg/reaction SOX8 (DSHB, PCRP-SOX8-2B9), 0.755 μg/reaction SOX8 (GeneTex, GTX129949), 0.5 μg/reaction SOX8 (DSHB, PCRP-SOX8-2D11), 0.6 μg/reaction SOX8 (Proteintech, 20627-1-AP), 0.5 μg/reaction SOX9 (DSHB, PCRP-SOX9-1A2), 0.5 μg/reaction SOX9 (DSHB, PCRP-SOX9-1E5), 0.618 μg/reaction SOX9 (Abcam, ab185966) and 0.5 μg/reaction SOX9 (Millipore Sigma, ab5535).

The SNAP-CUTANA K-MetStat Panel (EpiCypher, 19-1002k) was added to IgG, H3K4me3 and H3K27ac reactions. Final DNA was quantified using the Qubit 3 Fluorometer and the Qubit dsDNA HS Assay Kit (ThermoFisher Scientific, Q32854). Up to 5 ng of DNA per reaction was used for library preparation. The EpiCypher CUTANA CUT&RUN Library Prep protocol v1.5 was followed using the NEBNext Ultra II DNA Library Prep Kit for Illumina (NEB, E7645S). Provided adapters were diluted 1:25, per manufacturer's recommendation. Indexing was performed usingNEBNext Multiplex Oligos for Illumina (Dual Index Primers Set 1) (NEB, E7600S) with 15 cycles of index amplification. Libraries were sequenced at the Novogene Sequencing Core on a 10B lane of the NovaSeq X Plus system (Illumina).

### CUT&RUN data analysis

Raw FASTQ files were trimmed to remove adaptor sequences using the Trim Galore wrapper for cutadapt (Martin, 2011). Trimmed reads were aligned to the *Mus musculus* mm10 and *E. coli* K-12 MG1655 spike-in reference genome using Bowtie2 (Langmead and Salzberg, 2012). Duplicate reads and low-quality alignments were removed with Picard and Samtools prior to downstream analysis (Li et al., 2009; http://broadinstitute.github.io/picard/). Spike-in normalization was performed by scaling mouse-aligned read counts to the proportion of reads mapped to the *E. coli* genome. Peaks were called using SEACR in stringent mode with IgG controls used as background to identify high-confidence enriched regions (Meers et al., 2019). Motif enrichment analysis was performed using HOMER, which identified strong SOX motif enrichment specifically in CUT&RUN datasets generated with antibodies against SOX8 (ab3, GeneTex, GTX129949; ab5, Proteintech, 20627-1-AP) and SOX9 (ab4, Millipore Sigma, ab5535) (Heinz et al., 2010). To generate a unified set of Sox8- and Sox9-bound regions, replicate peak files from SEACR were intersected for each antibody and age, and overlapping intervals were merged to obtain high-confidence Sox8 peak sets for P2 and P17. Sox8 and Sox9 peaks were then combined at each age by reducing overlapping intervals to create merged Sox8 and Sox9 peak sets. Combined Sox8 and Sox9 peak sets were intersected with developmental ATAC-Seq DARs and Sox8 and Sox9 conditional knockout DARs in MG identified previously, to determine age-specific or chromatin accessibility changes associated with Sox binding. Peaks were annotated to genes and genomic features using ChIPseeker, with promoter, enhancer and dual-marked status determined based on overlap with H3K4me3 and H3K27ac CUT&RUN peaks (Yu et al., 2015).

## Supplementary Material



10.1242/develop.205508_sup1 Supplementary information

Table S1. ScRNA-Seq analysis of SOX8+SOX9 overexpression. Differentially expressed genes are shown for GFP-positive cells from retinas electroporated at E14 with either pCAGIG-SOX8+pCAGIGSOX9 or pCAGIG alone and harvested at P0. For RPCs, differential expression is shown for *SOX8* + *SOX9* vs. control. For oligodendrocyte lineage-like cells, results are shown for SOX8+SOX9 only, and differential expression calculated for OL-like cells vs. all cells analyzed.

Table S2. ScRNA-Seq analysis of P14 and P120 *Chx10-Cre;Sox9^lox/lox^* retina. Differentially expressed genes are shown for MG in mutant vs. age-matched wildtype controls.

Table S3. ScRNA/ATAC-Seq multiome data from *Glast-CreER;Sox8^lox/lox^;Sox9^lox/lox^;CAG-lsl-Sun1-GFP* retina. Tamoxifen was administered via daily intraperitoneal injections at P3-5 and GFP-positive MG isolated via FACS at P17 in parallel with *CAG-lsl-Sun1-GFP* controls. Differentially-expressed genes (DEG), Gene Ontology analysis of differentially-expressed genes, differentially-accessible chromatin regions (DAR), differentially accessible transcription factor binding motifs, and overlap between DEG and DARs in mutant MG are shown.

Table S4. ScRNA-Seq data from *Rax-CreER; Sox8^lox/lox^;Sox9^lox/lox^;CAG-lsl-Sun1-GFP* retina. Tamoxifen was administered via daily intraperitoneal injections at P3-5 and whole retinas harvested at and dissociated P90. Differentially expressed genes in MG are shown.

Table S5. CUT&RUN analysis of Sox8/9 and histone modifications associated with active transcription. Chromosomal regions detected using antibodies for Sox8, Sox9, both Sox8 and Sox9 in combination, H3K4me3, and H3K27Ac are shown for whole retina at P2 and P17. Transcription factor binding motifs enriched in targeted regions are also shown.

Table S6. List of antibodies and commercial assays used in the study. Columns identify the reagent, source, catalog number, and RRID identifier if applicable.

Table S7. Cell counts for histological markers. Data is shown for SOX8+SOX9 overexpression and matched controls.
