# Supplementary material for: Sox8 and Sox9 regulate differentiation and nuclear positioning of retinal Müller glia
Source: Development. 2026 Oct 13;153(19):dev205508. doi: 10.1242/dev.205508 (PMC13723407; doi:10.1242/dev.205508)
Supplement: Supplementary file 1 [file develop-153-205508-s1.pdf]

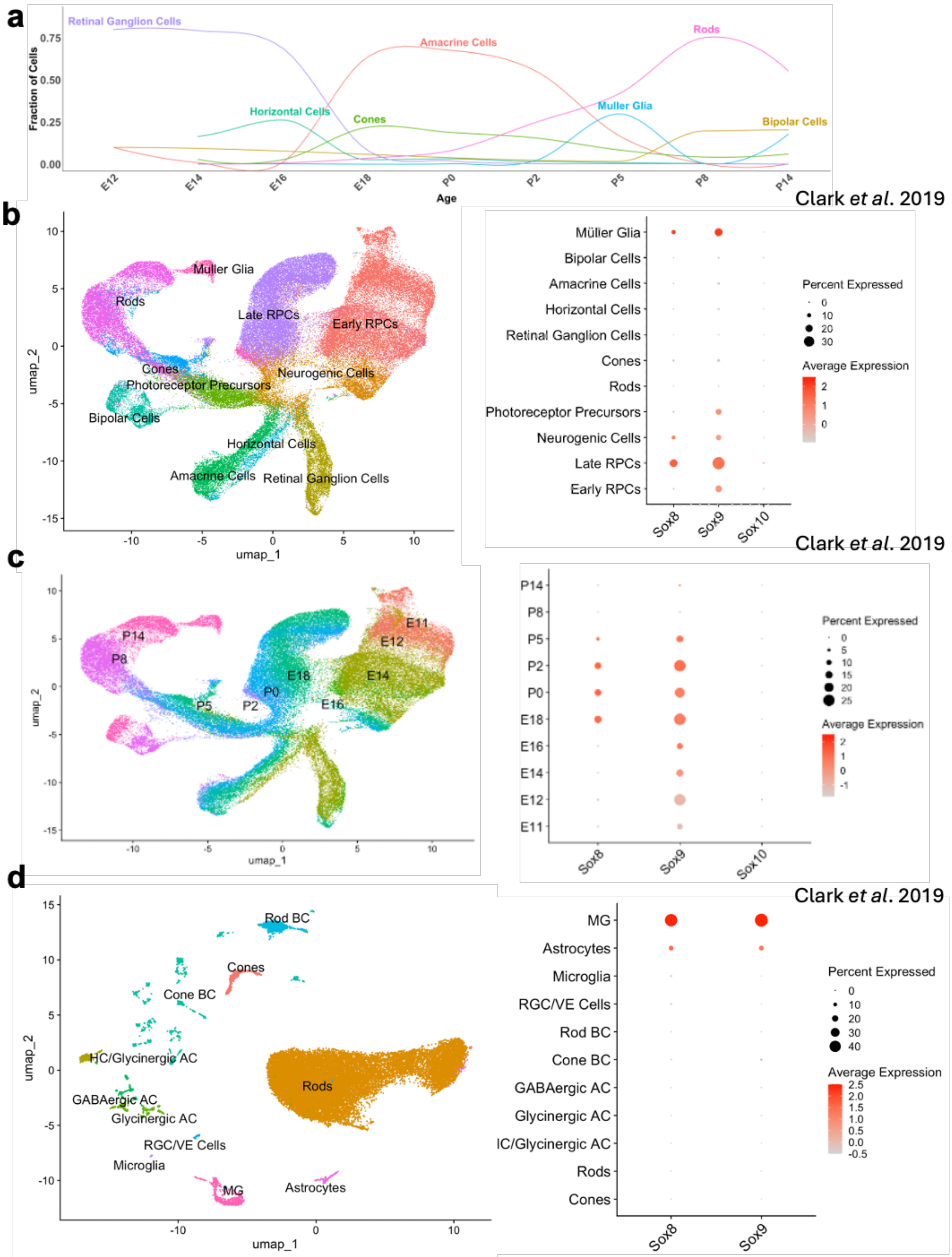


**Fig. S1. Expression of SoxE family transcription factors in the developing and mature mouse retina**. **(a)** Line graph showing the birth of major retinal cell types over the course of retinal development. **(b and c)** UMAP plot of scRNA-Seq data from developing mouse retina and dot plot showing cellular expression levels of SoxE family transcription factors in each major cell type **(b)** and time point **(c)**. **(d)** UMAP plot of scRNA-Seq data from adult mouse retina and dot plot showing cellular expression levels of *Sox8* and *Sox9* in each major cell type.

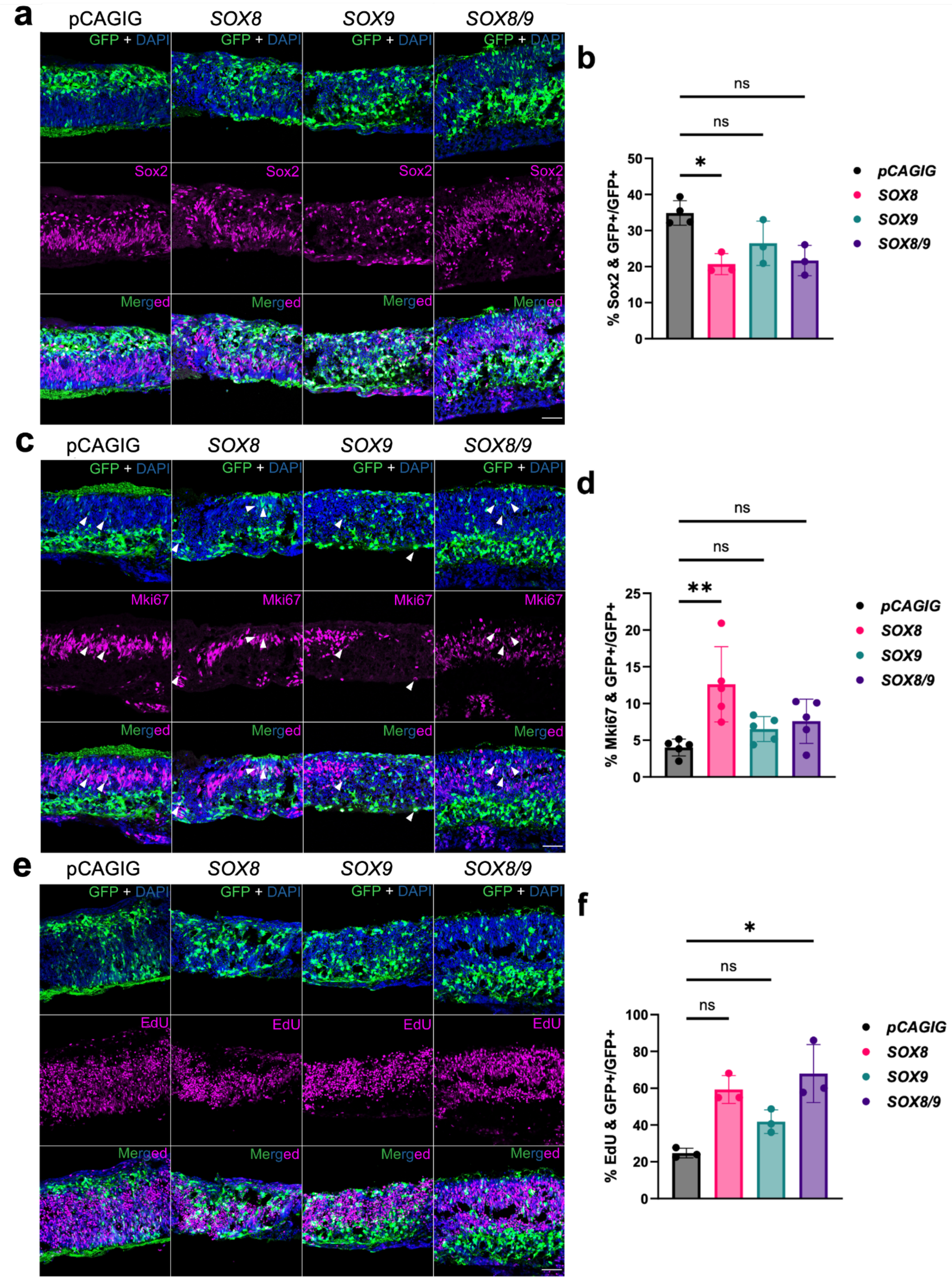
a
pCAGIG
SOX8
SOX9
SOX8/9
GFP + DAPI
Sox2
Merged
b
% Sox2 & GFP+/GFP+
ns
ns
*
pCAGIG
SOX8
SOX9
SOX8/9
c
Mki67
d
% Mki67 & GFP+/GFP+
**
e
EdU
f
% EdU & GFP+/GFP+

**Fig. S2. Overexpression of *SOX8* and *SOX9* in retinal explant cultures.**

**(a, c, e)** Representative immunostaining for GFP and **(a)** Sox2 (retinal progenitor cells and amacrine cells), **(c)** Mki67 (actively proliferating cells), and **(e)** EdU pulsed at E16 and chased until explant collection at P0. Scale bars, 50 μm. DAPI, 4′,6-diamidino-2-phenylindole. **(b, d, f)** Quantification of mean percentage ± SD of GFP-positive electroporated cells that co-label with **(b)** Sox2, **(d)** Mki67 or **(f)** EdU for each condition. White arrowheads indicate co-labeled GFP and Mki67-positive cells. Significance was determined via Kruskal-Wallis with Dunn's multiple comparisons: *$P < 0.0332$, **$P < 0.0021$, ns = not statistically significant. Each data point was calculated from an individual retina.

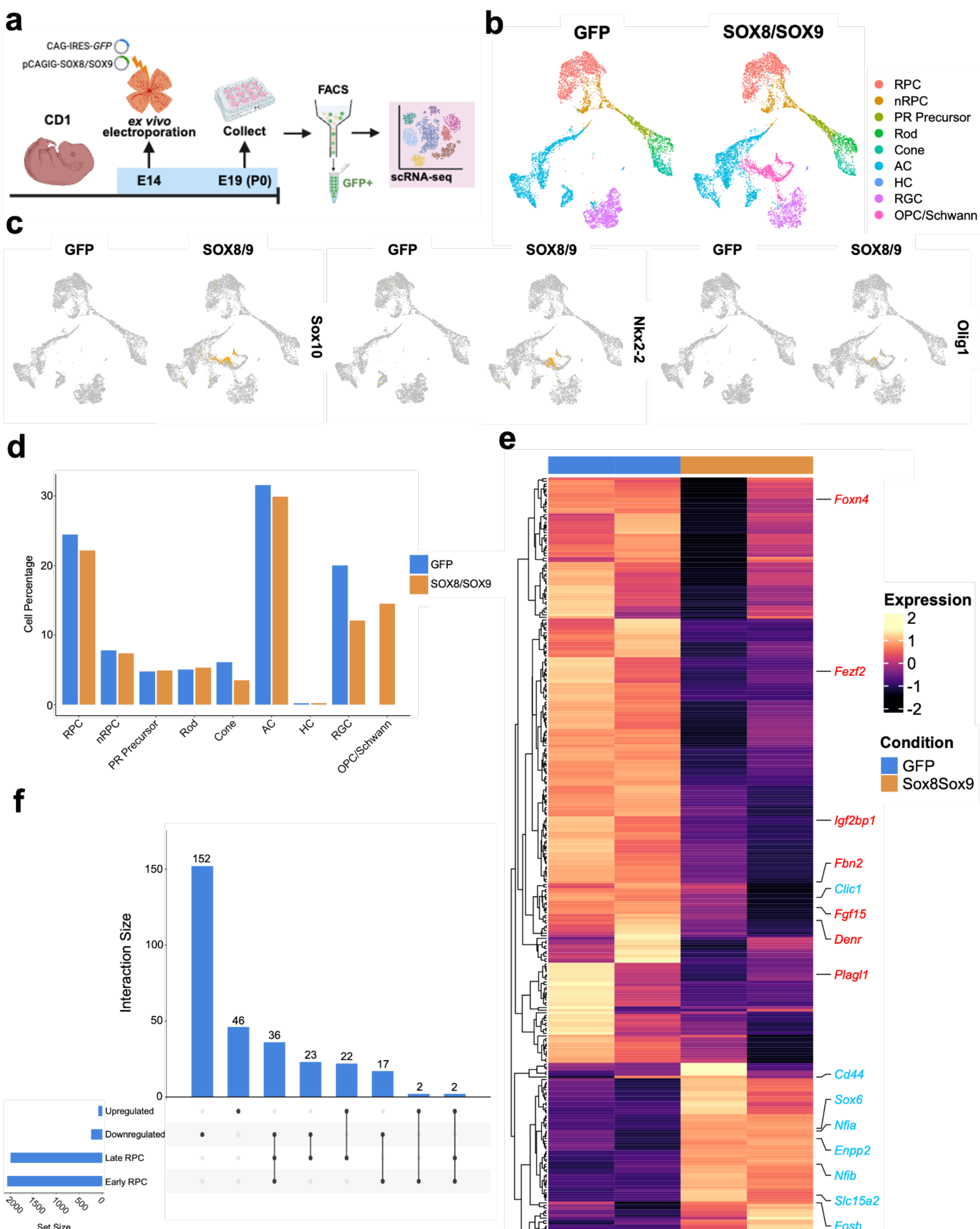


Development • Supplementary information

**Fig. S3. scRNA-seq analysis of *SOX8* and *SOX9* overexpression in retinal explant cultures.**

**(a)** Schematic of the experimental workflow. **(b)** UMAP plot of GFP (control) and SOX8/SOX9 overexpression conditions. **(c)** Feature plots showing expression of *Sox10*, *Nkx2-2*, and *Olig1* in the OPC/Schwann cell cluster. **(d)** Cell percentages for GFP and SOX8/SOX9 overexpression conditions. **(e)** Heatmap showing key differentially-expressed genes in RPCs. Red text indicates genes that are specific to early-stage RPCs, while blue text indicates genes that are specific to late-stage RPCs/MG. **(f)** Bar plot showing the number of differentially expressed genes in *SOX8/SOX9*-overexpressing RPCs that overlap with genes enriched in early and late-stage RPCs.

Development • Supplementary information

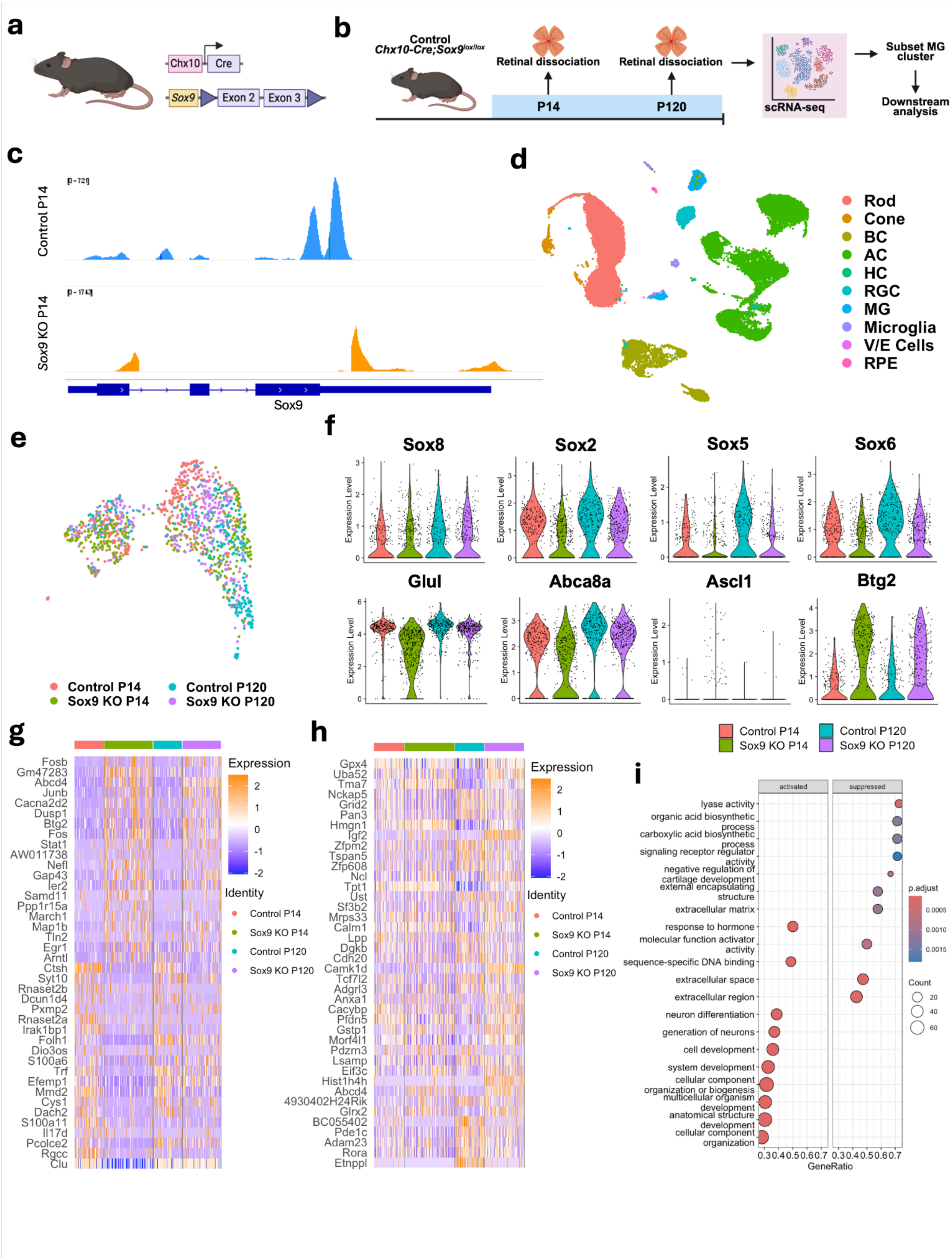


Development • Supplementary information

**Fig. S4. scRNA-Seq analysis of control and *Chx10;Sox9$^{lox/lox}$* Müller glia.** **(a)** Schematic of the transgenic constructs used to induce deletion of *Sox9* specifically in RPCs. Cre-mediated removal of Exons 2 and 3 of *Sox9* leads to premature stop codon formation and disrupts the expression of the Sox9 protein. **(b)** Schematic of the scRNA-Seq experimental pipeline. **(c)** scRNA-Seq read pileup showing efficient deletion of exon 2 of *Sox9* in P14 retinas. **(d)** UMAP plot of combined P14 and P120 control and *Sox9*-deficient retinas. **(e)** UMAP plot of subsetted MG showing the clustering of P14 and P120 control and *Sox9*-deficient MG. **(f)** Violin plots of MG markers (*Glul*, *Abca8a*), Sox transcription factors (*Sox8*, *Sox5*, *Sox6*, *Sox2*), neurogenic transcription factor *Ascl1*, and anti-proliferative factor *Btg2*. **(g, h)** Heatmap of top 20 upregulated and downregulated genes in P14 **(g)** and P120 **(h)** *Sox9*-deficient retinas. **(i)** Gene set enrichment analysis of significant differentially expressed genes in *Sox9*-deficient MG compared to control MG at P14.

Development • Supplementary information

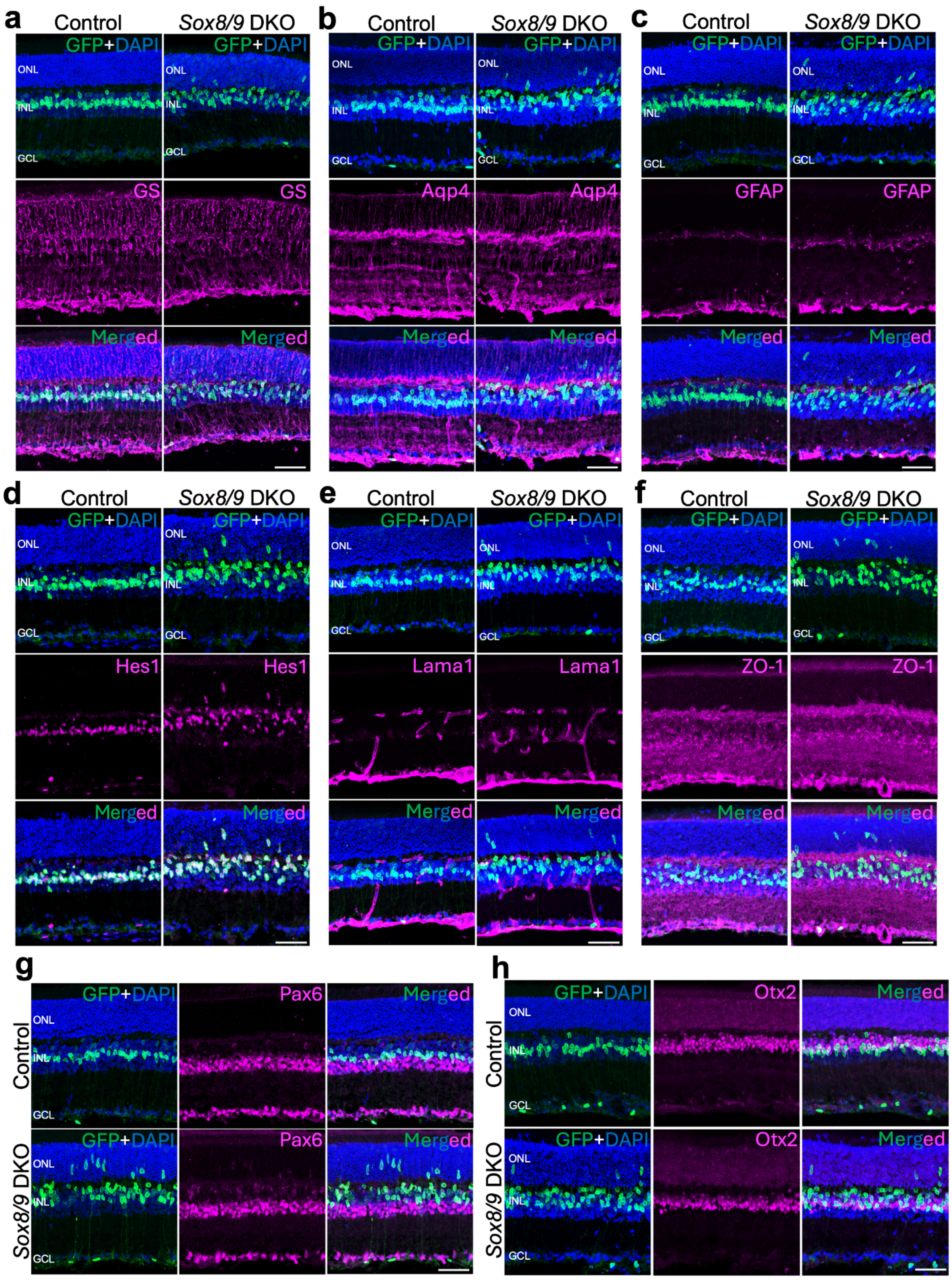


**Fig. S5. Immunohistochemical analysis of glial and neuronal markers in control and *Sox8/9*-deficient Müller glia.**

**(a-d)** Representative images of control and *Sox8/9* DKO retinas immunolabeled for GFP and glial markers, including **(a)** glutamine synthetase (GS), **(b)** Aqp4, **(c)** GFAP, and **(d)** Hes1. **(e-h)** Representative images of control and *Sox8/9* DKO retinas immunolabeled for GFP and **(e)** Lama1, **(f)** ZO-1, **(g)** Pax6, and **(h)** Otx2. ONL, outer nuclear layer; INL, inner nuclear layer; GCL, ganglion cell layer. DAPI, 4′,6-diamidino-2-phenylindole. Scale bar = 50μm.

Development • Supplementary information

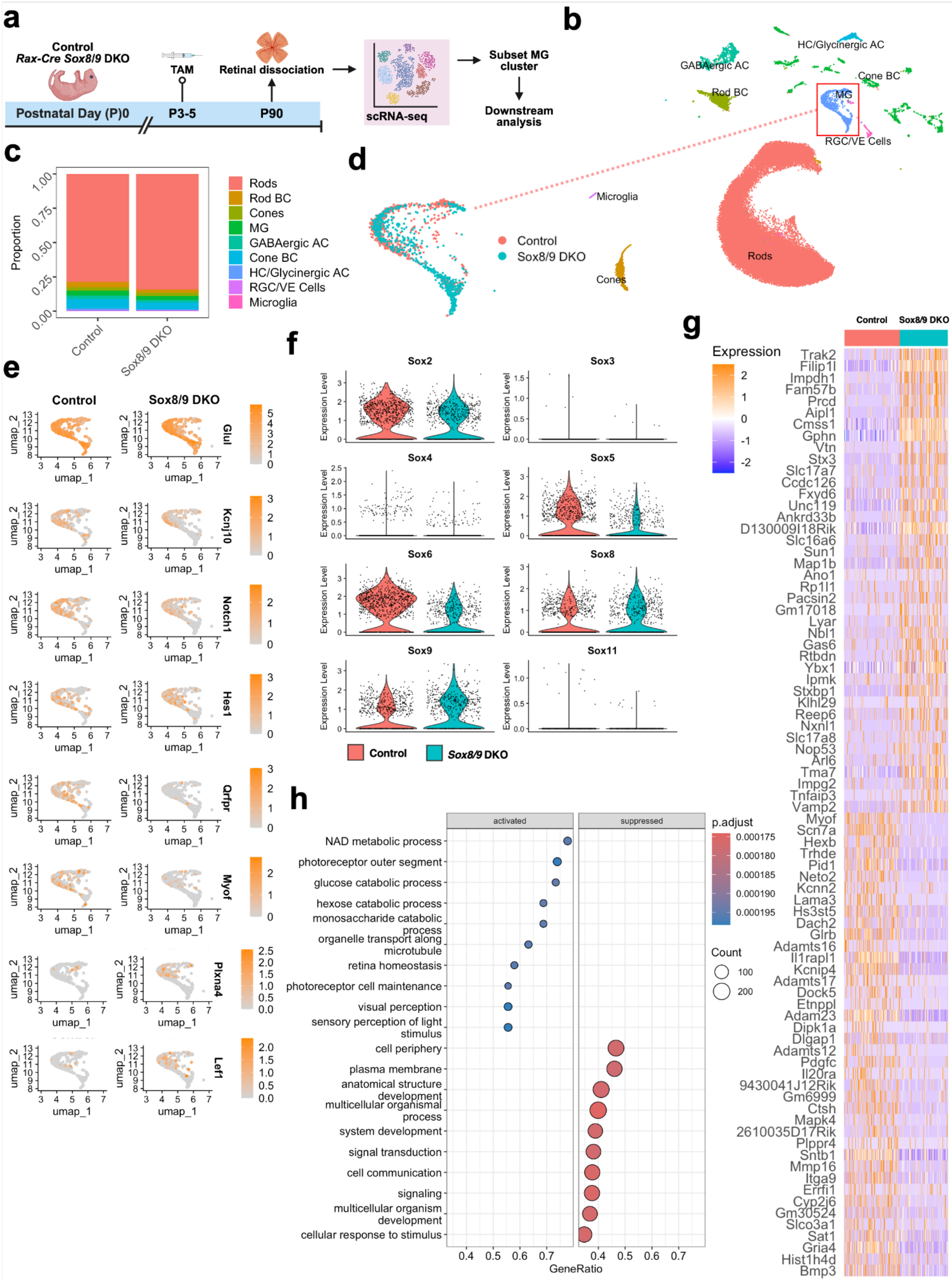


Development • Supplementary information

**Fig. S6. scRNA-Seq analysis of control and *Sox8/9* DKO mature Müller glia.**

**(a)** Schematic of the scRNA-Seq experimental pipeline. **(b)** UMAP plot of scRNA-Seq datasets showing the clustering of all major retinal cell types in control and *Sox8/9*-deficient retinas. **(c)** Stacked bar plots representing the proportion of cells in each cluster between control and *Sox8/9*-deficient retinas. **(d)** UMAP plot of scRNA-Seq datasets showing the clustering of all major retinal cell types in control and *Sox8/9*-deficient retinas. **(e)** Heatmap showing expression of the top 40 upregulated and downregulated genes in *Sox8/9*-deficient MG. **(f)** Feature plots highlighting differentially expressed genes associated with glial cell identity (*Glul*, *Kcnj10*) Notch signaling (*Notch1*, *Hes1*), cell migration (*Qrfpr*, *Myof*), cytoskeletal remodeling (Plxna4) and Wnt signaling (*Lef1*). **(g)** Violin plots showing decreased expression of Sox factors in *Sox8/9*-deficient MG. **(h)** Gene set enrichment analysis of significant differentially expressed genes in *Sox8/9*-deficient MG compared to control MG at P90.

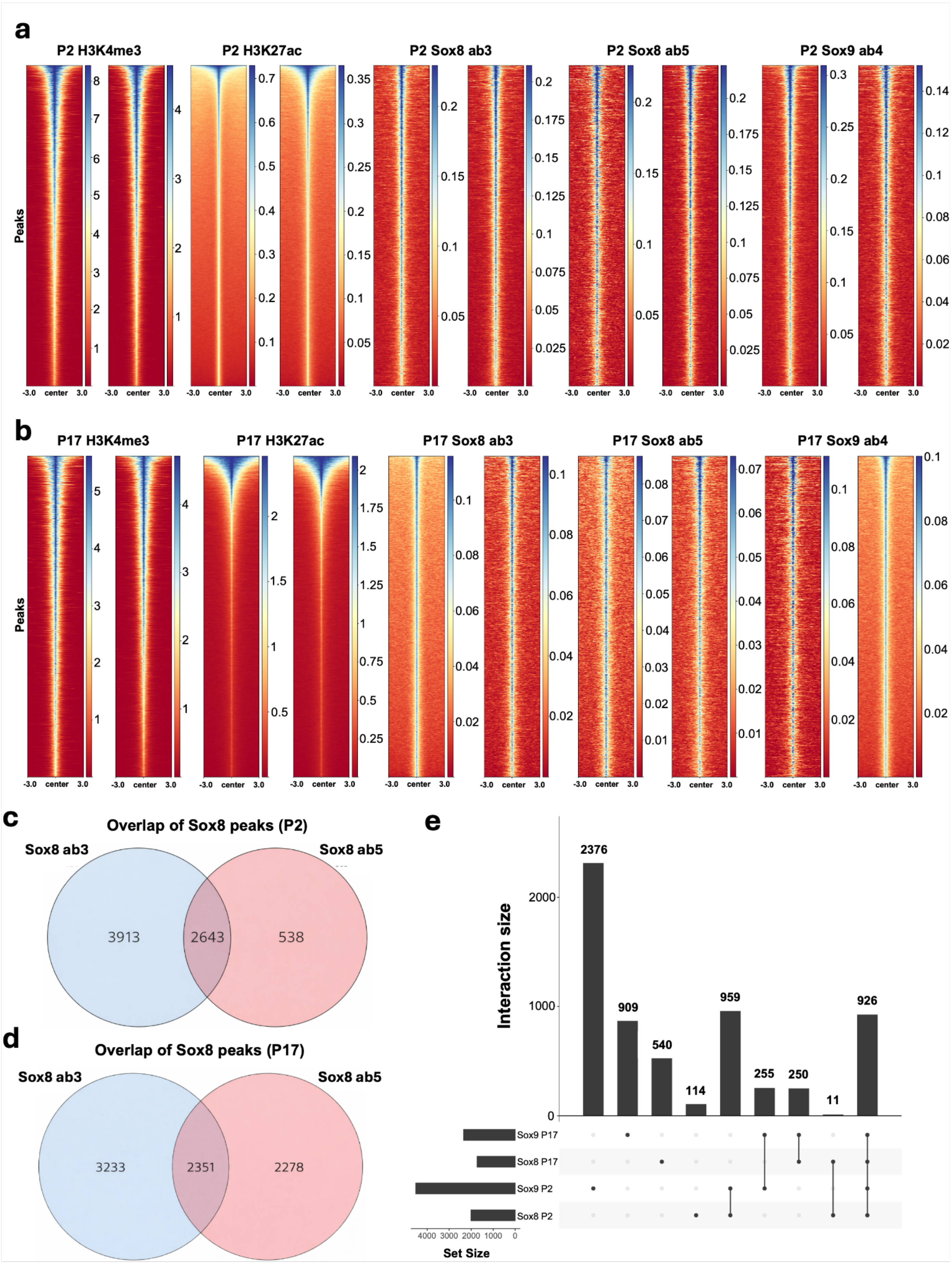


**Fig. S7. CUT&RUN analysis of Sox8 and Sox9.**
**(a-b)** Peak intensity plots for H3K4me3, H3K27ac, anti-Sox8 ab3, anti-Sox8 ab5 and anti-Sox9 ab4 at **(a)** P2 and **(b)** P17. Two replicate experiments were performed at both ages using each antibody. **(c-d)** Overlap of Sox8 peaks detected with ab3 and ab5 at **(c)** P2 and **(d)** P17. **(e)** Bar plot showing the number of overlapping peaks detected with Sox8 and Sox9 antibodies at P2 and P17.

Development • Supplementary information

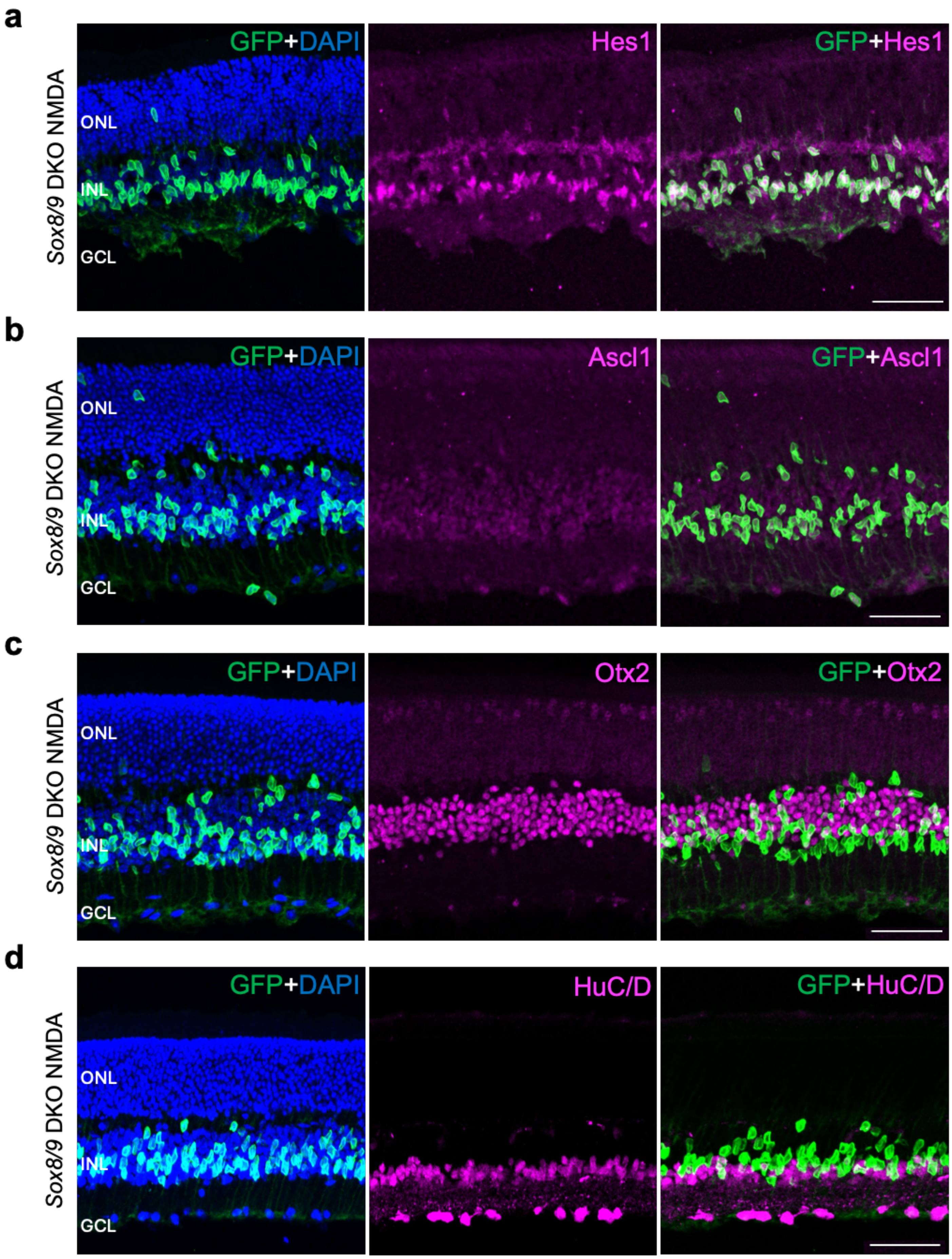


**Fig. S8. Selective loss of function of *Sox8/9* in mature MG does not induce neurogenesis with retinal injury.**

**(a-d)** Representative images of *Sox8/9* DKO retinas immunolabeled for glial **(a)** and neurogenic **(b-d)** markers, including **(a)** Hes1, **(b)** Ascl1, **(c)** Otx2, and **(d)** HuC/D. Scale bar = 50μm.

**Table S1. ScRNA-Seq analysis of *SOX8+SOX9* overexpression.** Differentially expressed genes are shown for GFP-positive cells from retinas electroporated at E14 with either pCAGIG-SOX8+pCAGIG-SOX9 or pCAGIG alone and harvested at P0. For RPCs, differential expression is shown for *SOX8 +SOX9* vs. control. For oligodendrocyte lineage-like cells, results are shown for SOX8+SOX9 only, and differential expression calculated for OL-like cells vs. all cells analyzed.

Available for download at
https://journals.biologists.com/dev/article-lookup/doi/10.1242/dev.205508#supplementary-data

**Table S2. ScRNA-Seq analysis of P14 and P120 *Chx10-Cre;Sox9$^{lox/lox}$* retina.** Differentially expressed genes are shown for MG in mutant vs. age-matched wildtype controls.

Available for download at
https://journals.biologists.com/dev/article-lookup/doi/10.1242/dev.205508#supplementary-data

**Table S3. ScRNA/ATAC-Seq multiome data from *Glast-CreER;Sox8$^{lox/lox}$;Sox9$^{lox/lox}$;CAG-lsl-Sun1-GFP* retina.** Tamoxifen was administered via daily intraperitoneal injections at P3-5 and GFP-positive MG isolated via FACS at P17 in parallel with *CAG-lsl-Sun1-GFP* controls. Differentially-expressed genes (DEG), Gene Ontology analysis of differentially-expressed genes, differentially-accessible chromatin regions (DAR), differentially accessible transcription factor binding motifs, and overlap between DEG and DARs in mutant MG are shown.

Available for download at
https://journals.biologists.com/dev/article-lookup/doi/10.1242/dev.205508#supplementary-data

**Table S4. ScRNA-Seq data from *Rax-CreER; Sox8$^{lox/lox}$;Sox9$^{lox/lox}$;CAG-lsl-Sun1-*** *GFP* retina. Tamoxifen was administered via daily intraperitoneal injections at P3-5 and whole retinas harvested at and dissociated P90. Differentially expressed genes in MG are shown.

Available for download at
https://journals.biologists.com/dev/article-lookup/doi/10.1242/dev.205508#supplementary-data

Development • Supplementary information

**Table S5. CUT&RUN analysis of Sox8/9 and histone modifications associated with active transcription.** Chromosomal regions detected using antibodies for Sox8, Sox9, both Sox8 and Sox9 in combination, H3K4me3, and H3K27Ac are shown for whole retina at P2 and P17. Transcription factor binding motifs enriched in targeted regions are also shown.

Available for download at
https://journals.biologists.com/dev/article-lookup/doi/10.1242/dev.205508#supplementary-data

**Table S6. List of antibodies and commercial assays used in the study.** Columns identify the reagent, source, catalog number, and RRID identifier if applicable.

Available for download at
https://journals.biologists.com/dev/article-lookup/doi/10.1242/dev.205508#supplementary-data

**Table S7. Cell counts for histological markers.** Data is shown for SOX8+SOX9 overexpression and matched controls.

Available for download at
https://journals.biologists.com/dev/article-lookup/doi/10.1242/dev.205508#supplementary-data
