# Supplementary material for: Sox8 and Sox9 regulate differentiation and nuclear positioning of retinal Müller glia
Source: Development. 2026 Oct 13;153(19):dev205508. doi: 10.1242/dev.205508 (PMC13723407; doi:10.1242/dev.205508)
Supplement: Supplementary file 2 [file develop-153-205508_review_history.pdf]

Development

Sox8 and Sox9 regulate differentiation and nuclear positioning of retinal Müller glia

Nicole Pannullo, Debarpita Datta, Clayton P. Santiago, Jared A. Tangeman, Lizhi Jiang, Leighton H. Duncan, Taqdees Gohar, Megan Gimmen and Seth Blackshaw
DOI: 10.1242/dev.205508

Editor: Debra Silver

Review timeline

Original submission:	13 January 2026
Editorial decision:	24 February 2026
First revision received:	9 July 2026
Accepted:	20 August 2026

Original submission

<u>First decision letter</u>

MS ID#: dev.205508

MS TITLE: Sox8 and Sox9 regulate differentiation and nuclear positioning of retinal Müller glia.

AUTHORS: Nicole Pannullo; Debarpita Datta; Clayton Santiago; Jared Tangeman; Lizhi Jiang; Leighton Duncan and Seth Blackshaw

Dear Dr Blackshaw,

I have now received all the referees' reports on the above manuscript, and have reached a decision. The referees' comments are appended below.

As you will see, the referees express considerable interest in your work, but have some significant criticisms and recommend a substantial revision of your manuscript before we can consider publication. The reviewers request additional experiments to clarify the differentiation phenotypes, as well new inclusion of controls and quantification. Please note reviewers also cite the need for additional validation (scRNA seq not being essential so alternatives are welcome). Moreover you will see requests for many text modifications/discussion points, including to ensure that all conclusions are appropriate. Please note you can also make use of the Study Limitations section in your revision.

If you are able to revise the manuscript along the lines suggested, I will be happy to receive a revised version of the manuscript. Your revised paper will be re-reviewed by one or more of the original referees, and acceptance of your manuscript will depend on your addressing satisfactorily the reviewers' major concerns. Please also note that Development will normally permit only one round of major revision. If it would be helpful, you are welcome to contact us to discuss your revision in greater detail. Please send us a point-by-point response indicating your plans for addressing the referees' comments, and we will look over this and provide further guidance.

Please attend to all of the reviewers' comments and ensure that you upload both a 'clean' version of your Word file, along with a highlighted version clearly showing where you have made changes in the revised manuscript. Please avoid using 'Tracked changes' in Word files as these are lost in PDF conversion. I should be grateful if you would also provide a point-by-point response detailing how

© 2026. Published by The Company of Biologists under the terms of the Creative Commons Attribution License (https://creativecommons.org/licenses/by/4.0/).

you have dealt with the points raised by the reviewers in the 'Response to Reviewers' box. If you do not agree with any of their criticisms or suggestions please explain clearly why this is so.

Reviewer 1

SUMMARY OF THE ADVANCE MADE IN THIS PAPER AND ITS POTENTIAL SIGNIFICANCE TO THE FIELD

In this study, Pannullo et al investigated the roles of Sox8 and Sox9 during retinal development using loss- and gain-of-function assays at late and embryonic stages, respectively. They found that overexpression of these genes suppressed early cell fates during the embryonic window while deletion of these genes in late progenitors did not affect gliogenesis but did cause changes in Müller glia nuclear position and subtle changes in glial gene expression. Conditional loss of Sox8/9 in mature Müller glia under damage conditions induced proliferation but did not increase the neurogenic potential. This is a very thorough study that makes use of multiple Cre lines to alter Sox8/9 expression, either individually or in combination, at multiple stages. High-throughput assays are used to define gene expression changes induced by the absence of Sox8 and/or Sox9 during the late stage of retinal development. This is combined with analysis of associated changes in chromatin accessibility, and interpretation is strengthened by defining histone modifications and Sox8/9 direct targets using CUT&RUN analysis. Overall, the work is of high quality and adds to knowledge of late temporal patterning and the role of Sox8/9 in regulating Müller glia.

SUGGESTIONS TO AUTHORS:
1) In the abstract, at the end, it states " These findings suggest that Sox8/9 are dispensable for gliogenesis and repression of neurogenic competence, but are essential for proper laminar positioning and maturation of retinal Müller glia." However, it is an overstatement to say that they are essential for ""…maturation of retinal Müller glia." After dKO of Sox8/9 the authors show that almost all markers of mature Müller glia are still expressed. The changes in the transcriptional profile of Müller glia are modest and primarily related to cell migration and adhesion and not necessarily related to the overall state of Müller glia maturation.
2) It would be helpful to include SoxE genes and Müller glia, under Keywords and eliminate some of the more general terms
3) Line 42 - Stolt et al 2003 reference is duplicated.
4) In line 109 (Figure 1), the authors imply that Sox8/9 overexpression suppresses the generation of early-born cell types and promotes late photoreceptor differentiation by the increase in Otx2+ cells, but this marker labels photoreceptor precursors, which can generate cone photoreceptors (an early cell type) (Lonfat et al., 2021). The authors should include additional markers to validate this shift in progenitor competence. The authors also observed increased proliferation with Sox8 and/or Sox9 overexpression. Could the reduction in early cell type genesis occur by the extension of the proliferation window of progenitors that prevents them from differentiating? Bulk RNA-seq from GFP+ cells in each condition could easily reveal which transcriptional changes the overexpression of either or both genes would induce in early RPCs.
5) There appears to be significant disorganization of retinal lamination after Sox 8, Sox9, or Sox8/9 electroporation ex vivo (Figure 1) as evident by significantly altered distribution of Rbpms and Otx2. This should be noted and discussed as it does not appear with pCAGIG electroporation.
6) In Figure S2C the image for Mik67 labelling seems a little off from what is being described - it doesn't look like an increase in co-labeled cells with Sox8 overexpression (in fact co-labeled cells are difficult to discern). Again, the retinas after Sox8 or Sox9 overexpression seem thinner and more disorganized. The selected image seems to have more Mik67 cells in the Sox8/9 combination, but none really overlapping with GFP. Why does the combination of Sox8/9 not increase the number of proliferating cells as Sox8 alone does (panel d)? That should be addressed in the discussion and better image examples should be provided.
7) In line 118, the authors state" Overall, these results may reflect transient stimulation of proliferation in neurogenic RPCs…" Why do they suggest that proliferation occurs in neurogenic RPCs rather than primary RPCs?
8) Is the reduction of Sox2+ cells in Figure S2a a direct result of Sox8/9 regulating the expression of this gene, or is it reflecting a reduction in RPCs as the authors suggest? The authors should discuss this.
9) In Figures 2 and 3 is the overall number of Sox2+ cells increased? In panels Figure 2E-F using both mouse models, does deletion of Sox genes affects the number of Sox2+ in the inner part of the INL (not MG)? This could be quickly quantified as a control.

© 2026. Published by The Company of Biologists under the terms of the Creative Commons Attribution License (https://creativecommons.org/licenses/by/4.0/).

10) In Figure S4 it seems that none of the displaced cells express the MG markers analyzed except Hes1, which can label progenitors during development. Could those cells be MG-derived progenitors? The authors reported that no GFP+ cells express Pax6 in the ONL, but in this image, some appear to express low levels of Pax6 (which marks progenitors). Even in the INL, some GFP+ cells seem to express Pax6. This should be addressed and discussed.
11) In line 195, the authors highlighted that "most strongly downregulated genes were enriched for regulators of cell adhesion (Pcdh10, Pcdh18, Alcam1), cell migration (Prss22, Kit,Qrfpr), or myosin-dependent motility (Myh6, Myof) (Fig. 4E, F)." but in line 199 says " Strongly upregulated genes included regulators of cell migration and/or synaptogenesis (Robo3, Plxna4), extracellular matrix (Hs6st3, Fbn2), or Wnt signaling (Lef1, Wnt5b) (Fig. 4H-J)." The cell migration process is highlighted as being down- and up-regulated. Are those genes working in different ways (inhibiting or promoting the process)? That should be explained, if that is the case.
12) In Figure S5 panel f, one would expect that Sox8 and Sox9 expression would be drastically reduced in dKO animals, but that doesn't seem to be the case. On line 224 the authors state that they (along with other Sox factors) are moderately downregulated, but in the violin plots they actually look upregulated. Is there an explanation for that? It would be helpful to add statistical information to the plots. I don't think this data helps to argue the changes, especially given the previous observations.
13) There is little explanation for the data in Figure S6. What information could be obtained from these results?
14) Figure 5 - P17 scATAC-Seq data generated for this study is not represented in the manuscript. For example in panel F - How were the peaks associated with MG defined?
15) Lines 254-257 is missing reference to a figure or table to support the statement.
16) For Figure 6 the authors evaluated the influence of Sox8/9 deletion only in an injury model; what happens in uninjured adult MG when these genes are deleted?
17) GFP+ cells in the control condition under damage show a sparse distribution, with cells close to the ONL, similar to Sox8cKO NMDA condition and different from Sox9cKO NMDA and SOX8/9 DKO uninjured retinas. Any reason for this difference? Could it suggest that Sox9 regulates MG migration?
18) The Sox8cKO NMDA image shows an Edu+ cell that is not GFP+. What does that mean? How often is that observed?
19) When deleting both genes in mature MG, this doesn't seem to promote migration, as no cells are observed in the ONL. That should be discussed.
20) In the Discussion on line 286: The authors state that overexpression was sufficient to bias toward a later-born fate, but they only evaluated the Otx2 marker, which could be expressed by precursors that can generate cones (early cell type). Lines 288 and 300 have similar statements.
21) Line 345 on data availability refers to bulk RNA-seq data, but only scRNA-seq data were shown in this work. Are the P17 scATAC-seq data also deposited?

Reviewer 2

SUMMARY OF THE ADVANCE MADE IN THIS PAPER AND ITS POTENTIAL SIGNIFICANCE TO THE FIELD

This manuscript investigates the role of Sox8 and Sox9 in mouse retinal development and regeneration using gain and loss of function approaches combined with single-cell multiomic profiling. Based on prior studies, the authors hypothesized that Sox8/9 expression in late progenitors or mature Müller glia could repress neurogenic competence. However, conditional loss of Sox8/9 in late-stage retinal precursors did not alter retinal cell fate, contrasting with earlier reports suggesting that these factors promote gliogenesis and suppress neurogenesis. Instead, the authors observed a modest reduction in mature Müller glia markers and a displacement of Müller glia nuclei toward the outer retina. In an injury paradigm, Sox8/9 deletion in Müller glia also failed to induce neurogenic competence. The authors conclude that although Sox8/9 influence gene expression and nuclear positioning in Müller glia, they are dispensable for retinal gliogenesis.

Overall, the manuscript is well-written and illustrated, with high-quality genetic approaches and multiomic profiling. It provides new insights to the field, particularly by refining prior models regarding the role of SoxE transcription factors in Müller glia specification/maintenance. However, several aspects of the experimental design and data interpretation warrant clarification, and some conclusions appear overstated.

© 2026. Published by The Company of Biologists under the terms of the Creative Commons Attribution License (https://creativecommons.org/licenses/by/4.0/).

SUGGESTIONS TO AUTHORS

Major comments:

1) The timing of Sox8/9 deletion in late-stage retinal precursors raises an important concern regarding the interpretation of these experiments. Müller glia are generated approximately between P2 and P8 (as summarized in Fig. S1A), yet tamoxifen was administered between P3 and P5. Could the authors exclude the possibility that this timing, occurring after the onset of Müller glia specification, is insufficient to impact fate determination? This could explain the divergence from previous studies, such as Muto et al. and Poché et al., which targeted earlier developmental windows. Although the authors acknowledge a timing difference in the Discussion, the conclusion (for instance in the Abstract, line 20) that Sox8/9 are dispensable for Müller cell specification appears overstated. Given the experimental design, the data support conclusions about maintenance of glial identity but probably not about specification.
2) The loss of function phenotype is described as "displacement of MG nuclei into the outer nuclear layer." However, in Fig. 2F, while displacement is evident, it is rarely into the ONL itself. The description should more accurately reflect the observed phenotype (for instance "displacement toward the outer nuclear layer").
3) In the injury paradigm, the main loss of function phenotype is Müller glia nuclear displacement. This phenotype is not discussed in the context of interkinetic nuclear migration (INM), despite the reported increase in proliferation. Could the observed displacement reflect cell cycle re-entry rather than a defect in laminar positioning per se?
4) Proliferation was assessed in the overexpression condition and in the injury model upon loss of function, but not in the loss of function experiments targeting late-stage retinal progenitor cells. Given the observed nuclear displacement, it would be informative to assess proliferation in this condition as well. This would help establish a potential link between nuclear displacement and proliferation.
5) Along the same line, the authors highlight Sox8/9 target genes associated with cell migration. However, if Müller glia nuclear displacement is related to INM, one might expect enrichment of cell cycle-related targets among Sox8/9-regulated genes. Have the authors examined this possibility? Clarifying whether cell cycle regulators are significantly represented in the dataset would strengthen the interpretation.
6) In Fig. 1C, a marked displacement of Rbpms$^+$ cells is visible. This phenotype is not discussed although it would deserve explicit commentary to clarify its significance.
7) Controls at P16 should be included in Figure 3D and 3I to allow a direct comparison with the control conditions.
8) ANOVA is applied here to small sample sizes (n=3-4 retinas per condition). A non-parametric test such as Kruskal-Wallis would be more appropriate for these sample sizes.

Minor comments:

1) The reported increase in Mki67+& GFP+/GFP+ cells is not readily apparent in Fig. S2C. Adding arrowheads highlighting double-positive cells would improve clarity.
2) Page 1, lines 41-42: Stolt et al. is cited twice in the same sentence.
3) Some figures show photoreceptors oriented up, while others show them down.

<u>Reviewer 3</u>

In their manuscript titled "Sox8 and Sox9 regulate differentiation and nuclear positioning of retinal Müller glia" Pannullo et al. describe the molecular and cellular dissection of the role of key retinal transcription factors in retinal development. They show that Sox8 and Sox9 are expressed in late-stage RPCs and retained in Müller glia from scRNA-seq data. Square wave electroporation of Sox8, Sox9 or the combination had a subtle effect on proliferation and cell fate specification in E14.5 retinal explants as measured by co-localization of GFP and immunofluorescence. Conditional knockout of Sox8, Sox9 or both led to displacement of Müller glial cell nuclei as measured by Sox2 immunoreactivity and other markers. Importantly, there did not appear to be any reactive gliosis that can lead to Müller glial cell displacement. Multiome analysis of purified Sox8/9 deficient Müller glia indicated dramatic changes in gene expression and cut&run showed the landscape of TF binding across the genome. Finally, they deleted Sox8/9 in adult mice and showed there was no increase in neurogenic competence.

© 2026. Published by The Company of Biologists under the terms of the Creative Commons Attribution License (https://creativecommons.org/licenses/by/4.0/).

1) For the E14.5 electroporation, the scoring of GFP and immunostaining can be challenging in tissue sections. To overcome this limitation and provide more rigorous data on the changes in cell fate with ectopic expression, the manuscript would benefit from dissociated cell staining and scoring. It is possible to visualize the GFP and the immunostaining and determine precise proportions.
2) To independently validate those results in (1) it would also be useful to perform scRNA-seq on the purified cells from the electroporation.
3) The omics analysis of the knockout were elegant but it wasn't clear what we learned from those studies. What are the key targets of Sox8/9 in Müller glia? What exactly are the consequences of loss of the TFs? There were no downstream functional studies to validate any of the proposed targets or pathways. It may not be feasible to do more experiments but a discussion would be beneficial.

First revision

Author response to reviewers' comments

We thank the Reviewers for their constructive and insightful comments on the manuscript. We have extensively revised the manuscript, adding new data that includes scRNA-Seq analysis of the effects of overexpression of Sox8/9 in early-stage retinal progenitors. We address each individual point that has been raised below, with responses highlighted in blue font.

Comments from the Reviewers:
Reviewer 1: SUMMARY OF THE ADVANCE MADE IN THIS PAPER AND ITS POTENTIAL SIGNIFICANCE TO THE FIELD

In this study, Pannullo et al investigated the roles of Sox8 and Sox9 during retinal development using loss- and gain-of-function assays at late and embryonic stages, respectively. They found that overexpression of these genes suppressed early cell fates during the embryonic window while deletion of these genes in late progenitors did not affect gliogenesis but did cause changes in Müller glia nuclear position and subtle changes in glial gene expression. Conditional loss of Sox8/9 in mature Müller glia under damage conditions induced proliferation but did not increase the neurogenic potential. This is a very thorough study that makes use of multiple Cre lines to alter Sox8/9 expression, either individually or in combination, at multiple stages. High- throughput assays are used to define gene expression changes induced by the absence of Sox8 and/or Sox9 during the late stage of retinal development. This is combined with analysis of associated changes in chromatin accessibility, and interpretation is strengthened by defining histone modifications and Sox8/9 direct targets using CUT&RUN analysis. Overall, the work is of high quality and adds to knowledge of late temporal patterning and the role of Sox8/9 in regulating Müller glia.

We thank the Reviewer for their positive assessment of the work.
SUGGESTIONS TO AUTHORS:
1) In the abstract, at the end, it states " These findings suggest that Sox8/9 are dispensable for gliogenesis and repression of neurogenic competence, but are essential for proper laminar positioning and maturation of retinal Müller glia." However, it is an overstatement to say that they are essential for "…maturation of retinal Müller glia." After dKO of Sox8/9 the authors show that almost all markers of mature Müller glia are still expressed. The changes in the transcriptional profile of Müller glia are modest and primarily related to cell migration and adhesion and not necessarily related to the overall state of Müller glia maturation.

We agree with the Reviewer that loss of *Sox8/9* does not lead to a failure of Müller glial terminal differentiation per se, but do not fully agree with this interpretation since Sox8/9-deficient cells significantly downregulate classic markers of mature resting Müller glia such as *Glul*, *Kcnj10*, *Slc1a3*, as well as other more recently identified genes in this category such as *Rrh* and *Frzb* (Fig. 4C, Table ST3). This implies that, while mutant cells retain their glial identity, expression of

© 2026. Published by The Company of Biologists under the terms of the Creative Commons Attribution License (https://creativecommons.org/licenses/by/4.0/).

markers of terminal differentiation is compromised. To clarify this point, we have added the following text to the Abstract:

"but caused radial displacement of glial nuclei into the outer retina and reduced expression of multiple markers of mature Müller glia".

And:
"These findings suggest that *Sox8/9* are dispensable for gliogenesis and repression of neurogenic competence, but are essential for proper laminar positioning and full terminal differentiation of retinal Müller glia."

2) It would be helpful to include SoxE genes and Müller glia, under Keywords and eliminate some of the more general terms

This is now included.

3) Line 42 - Stolt et al 2003 reference is duplicated.

This has been corrected.

4) In line 109 (Figure 1), the authors imply that Sox8/9 overexpression suppresses the generation of early-born cell types and promotes late photoreceptor differentiation by the increase in Otx2+ cells, but this marker labels photoreceptor precursors, which can generate cone photoreceptors (an early cell type) (Lonfat et al., 2021). The authors should include additional markers to validate this shift in progenitor competence. The authors also observed increased proliferation with Sox8 and/or Sox9 overexpression. Could the reduction in early cell type genesis occur by the extension of the proliferation window of progenitors that prevents them from differentiating? Bulk RNA-seq from GFP+ cells in each condition could easily reveal which transcriptional changes the overexpression of either or both genes would induce in early RPCs.

This point is correct, and is now addressed by inclusion of scRNA-Seq data from P0 GFP+ cells electroporated with *SOX8+SOX9* at E14, which is shown in Fig. S3 and Table ST1. This confirms that RGC generation is reduced and genes specific to late-stage RPCs, including Nfia and Nfib, are upregulated. Unexpectedly, this also reveals that Sox8/9 overexpression induces formation of an oligodendrocyte/OPC-like cell type, probably through induction of multiple transcription factors that are known to regulate oligodendrocyte specification and/or differentiation, including *Sox10*, *Nkx2-2*, *Olig1/2*, and *Myrf*. An overview of these findings is included in the Discussion. It is unclear why *SOX8/9* can confer competence for oligodendrocyte generation to retinal progenitors, but lack this ability in other CNS regions such as dorsal spinal cord. We realize that this is a very surprising result, which in some ways overshadows the main topic of the manuscript, and is obviously an exciting topic for further study. However, we firmly believe that it is more appropriate to include this work in a future manuscript, and maintain the current focus of what is already a very data-dense study.

5) There appears to be significant disorganization of retinal lamination after Sox 8, Sox9, or Sox8/9 electroporation ex vivo (Figure 1) as evident by significantly altered distribution of Rbpms and Otx2. This should be noted and discussed as it does not appear with pCAGIG electroporation.

This is now discussed as follows:
"Disordered lamination, particularly of Rbpms-positive retinal ganglion cells, was also frequently observed following overexpression of *SOX8* and *SOX9*, either individually or in combination, consistent with disrupted expression of genes controlling cell adhesion and lamination in RPCs."

6) In Figure S2C the image for Mik67 labelling seems a little off from what is being described - it doesn't look like an increase in co-labeled cells with Sox8 overexpression (in fact co-labeled cells are difficult to discern). Again, the retinas after Sox8 or Sox9 overexpression seem thinner and more disorganized. The selected image seems to have more Mik67 cells in the Sox8/9 combination, but none really overlapping with GFP. Why does the combination of Sox8/9 not increase the number of proliferating cells as Sox8 alone does (panel d)? That should be addressed in the

© 2026. Published by The Company of Biologists under the terms of the Creative Commons Attribution License (https://creativecommons.org/licenses/by/4.0/).

discussion and better image examples should be provided.

Cells co-expressing Mki67 and GFP are now indicated with white arrows.

7) In line 118, the authors state" Overall, these results may reflect transient stimulation of proliferation in neurogenic RPCs..." Why do they suggest that proliferation occurs in neurogenic RPCs rather than primary RPCs?

The word “neurogenic” has been removed, with the revised text reading as follows:

“Overall, these results may reflect transient stimulation of proliferation in RPCs followed by cell cycle exit and depletion of primary RPCs, again consistent with a shift towards later-stage temporal identity.”

8) Is the reduction of Sox2+ cells in Figure S2a a direct result of Sox8/9 regulating the expression of this gene, or is it reflecting a reduction in RPCs as the authors suggest? The authors should discuss this.

ScRNA-Seq analysis (Fig. S3d) does indeed demonstrate a modest reduction in the relative fraction of RPCs following overexpression of Sox8/9. We do not observe any overall change in cellular levels of Sox2 expression.

9) In Figures 2 and 3 is the overall number of Sox2+ cells increased? In panels Figure 2E-F using both mouse models, does deletion of Sox genes affects the number of Sox2+ in the inner part of the INL (not MG)? This could be quickly quantified as a control.

There is no significant change in the overall number of Sox2+ cells following deletion of Sox8 and/or Sox9 at any of the developmental timepoints assessed in either model (See Reviewer Fig. 1).

10) In Figure S4 it seems that none of the displaced cells express the MG markers analyzed except Hes1, which can label progenitors during development. Could those cells be MG-derived progenitors? The authors reported that no GFP+ cells express Pax6 in the ONL, but in this image, some appear to express low levels of Pax6 (which marks progenitors). Even in the INL, some GFP+ cells seem to express Pax6. This should be addressed and discussed.

MG normally express low levels of Pax6, consistent with the levels observed in Fig. S5g (c.f. PMID: 21500284). We do not observe the high levels of Pax6 expression in the mutant ONL that are characteristic of either mature amacrine and ganglion cells (as can be seen in Fig. S5g, formerly Fig.S4g) or of RPCs. We have modified the text to clarify this point as follows:

“we do not observe any displaced cells towards or in the ONL expressing the high levels of Pax6 characteristic of amacrine and ganglion cells (Fig. S5g)”.

11) In line 195, the authors highlighted that "most strongly downregulated genes were enriched for regulators of cell adhesion (Pcdh10, Pcdh18, Alcam1), cell migration (Prss22, Kit,Qrfpr), or myosin-dependent motility (Myh6, Myof) (Fig. 4E, F)." but in line 199 says " Strongly upregulated genes included regulators of cell migration and/or synaptogenesis (Robo3, Plxna4), extracellular matrix (Hs6st3, Fbn2), or Wnt signaling (Lef1, Wnt5b) (Fig. 4H-J)." The cell migration process is highlighted as being down- and up-regulated. Are those genes working in different ways (inhibiting or promoting the process)? That should be explained, if that is the case.

Genes linked to regulation of tumor cell migration are broadly upregulated, while those linked to regulation of neuronal migration are generally downregulated. This has been clarified in the text as follows:
“The most strongly downregulated genes were enriched for regulators of cell adhesion (*Pcdh10*, *Pcdh18*, *Alcam1*), tumor cell migration (*Prss22*, *Kit*, *Qrfpr*), or myosin-dependent motility (*Myh6*, *Myof*) (Fig. 4E,F). Several other Sox family members, including *Sox5* and *Sox6*, were also strongly downregulated, with *Sox2* also showing a modest reduction (Fig. 4G). Strongly upregulated genes included regulators of neuronal cell migration and synaptogenesis (*Robo3*, *Plxna4*), extracellular

© 2026. Published by The Company of Biologists under the terms of the Creative Commons Attribution License (https://creativecommons.org/licenses/by/4.0/).

matrix (*Hs6st3*, *Fbn2*), or Wnt signaling (*Lef1*, *Wnt5b*) (Fig. 4H-J)."

12) In Figure S5 panel f, one would expect that Sox8 and Sox9 expression would be drastically reduced in dKO animals, but that doesn't seem to be the case. On line 224 the authors state that they (along with other Sox factors) are moderately downregulated, but in the violin plots they actually look upregulated. Is there an explanation for that? It would be helpful to add statistical information to the plots. I don't think this data helps to argue the changes, especially given the previous observations.

As is the case with *Nfia/b/x* conditional mutants (Lyu, et al. 2021), the total levels of *Sox8/9* mRNA levels are not substantially downregulated following conditional loss of function. However, when scRNA-Seq reads from wildtype and *Sox8/9* DKO MG data are plotted using sashimi, we observe that *Sox8* mRNA is deleted downstream of exon 2 (see Reviewer Fig. 2A), and that while appropriate splicing patterns are preserved for a subset of *Sox9* mRNAs, that mutant samples show transcriptional readthrough upstream of exon 2 and the emergence of novel splice forms that omit virtually the entire coding sequence of the gene (see Reviewer Fig. 2B). This matches our immunohistochemical data, where virtually all MG lose Sox8 and ~20% lose Sox9 expression.

13) There is little explanation for the data in Figure S6. What information could be obtained from these results?

We have now added the following discussion of these results (Note that Figure S6 is now Figure S7):
"The great majority of Sox8/9 peaks associated with DARs present in P2 RPCs overlapped with H3K27ac, and in P17 MG with both H3K27ac and H3K4me3, indicating that they represent active promoter or enhancer elements (Fig. 5F,G; Fig. S7a,b). Substantial overlap was detected between peaks detected at P2 and P17 using two independent monoclonal antibodies against Sox8 (Fig. S7c,d), and between Sox8 and Sox9 peaks at both ages (Fig. S7e)."

14) Figure 5 - P17 scATAC-Seq data generated for this study is not represented in the manuscript. For example in panel F - How were the peaks associated with MG defined?

This is now described as follows:
"The data was integrated with both previously published scATAC-Seq data from P2 retina (Lyu et al. 2021) and P17 retinal scATAC-Seq data obtained from multiomic analysis both control and Sox8/9 DKO MG (Fig. 4A)."

15) Lines 254-257 is missing reference to a figure or table to support the statement.

We have now added a reference to Table ST5.

16) For Figure 6 the authors evaluated the influence of Sox8/9 deletion only in an injury model; what happens in uninjured adult MG when these genes are deleted?

We do not observe any proliferation in the absence of injury in *Sox8/9*-deficient MG. This data is presented in Fig. 6B, which shows images of EdU/GFP labeling for all genotypes and conditions, and Fig. 6C, where EdU/GFP double-positive cells are quantified.

17) GFP+ cells in the control condition under damage show a sparse distribution, with cells close to the ONL, similar to Sox8cKO NMDA condition and different from Sox9cKO NMDA and SOX8/9 DKO uninjured retinas. Any reason for this difference? Could it suggest that Sox9 regulates MG migration?

NMDA injury has been known for some time to induce radial displacement of MG nuclei in wildtype animals (c.f. Lebrun-Julien, et al. *J Neurosci* 2009). However, in contrast to the levels of EdU incorporation quantified in Fig. 6D, this is quite variable, and does not show consistent differences across genotypes.

18) The Sox8cKO NMDA image shows an Edu+ cell that is not GFP+. What does that mean? How often is that observed?

© 2026. Published by The Company of Biologists under the terms of the Creative Commons Attribution License (https://creativecommons.org/licenses/by/4.0/).

These cells are rare, and found only in NMDA-treated samples. While some may represent MG that did not efficiently remove the stop cassette from the Sun1-GFP reporter, most likely are dividing microglia, as suggested both by the fact that they are occasionally seen in NMDA- treated control samples and are essentially restricted to the OPL (c.f. Todd, et al. *J. Neuroinflamm*. 2019).

19) When deleting both genes in mature MG, this doesn't seem to promote migration, as no cells are observed in the ONL. That should be discussed.

While migration of MG nuclei into the ONL is not observed when Sox8/9 are deleted at P21, we do observe displacement of MG nuclei towards the OPL in the absence of excitotoxic injury, as can be seen in Fig. 6B, where this is clearly observed for Sox9-negative, Sox2-positive cells. This is now mentioned in the legend for Fig. 6B as follows:

"White arrowheads indicate co-labeled GFP and Sox2-positive cells that are Sox9-negative, which are radially displaced towards the OPL relative to Sox9-positive MG in the same section."

and discussed in the text as follows:

"Sox8/9-deficient MG nuclei are radially displaced towards the OPL (Fig. 6B), although in contrast to loss of function at P3-5 (Fig. 3), no GFP-positive nuclei are observed within the ONL itself in the absence of injury."

20) In the Discussion on line 286: The authors state that overexpression was sufficient to bias toward a later-born fate, but they only evaluated the Otx2 marker, which could be expressed by precursors that can generate cones (early cell type). Lines 288 and 300 have similar statements.

The Reviewer is indeed correct that Otx2 expression could represent cones in this context. However, this is not supported by scRNA-Seq analysis (now included in Fig. S3), and in any case cones are consistently born later than RGCs, which is the cell type whose generation is most clearly repressed by Sox8/9 overexpression (Fig. S3). The new scRNA-Seq data has in any case led us to reinterpret some of these conclusions, as discussed previously.

21) Line 345 on data availability refers to bulk RNA-seq data, but only scRNA-seq data were shown in this work. Are the P17 scATAC-seq data also deposited?

This wording was in error, and was indeed meant to refer to scRNA-Seq data. It has been corrected.

Reviewer 2: SUMMARY OF THE ADVANCE MADE IN THIS PAPER AND ITS POTENTIAL SIGNIFICANCE TO THE FIELD

This manuscript investigates the role of Sox8 and Sox9 in mouse retinal development and regeneration using gain and loss of function approaches combined with single-cell multiomic profiling. Based on prior studies, the authors hypothesized that Sox8/9 expression in late progenitors or mature Müller glia could repress neurogenic competence. However, conditional loss of Sox8/9 in late-stage retinal precursors did not alter retinal cell fate, contrasting with earlier reports suggesting that these factors promote gliogenesis and suppress neurogenesis. Instead, the authors observed a modest reduction in mature Müller glia markers and a displacement of Müller glia nuclei toward the outer retina. In an injury paradigm, Sox8/9 deletion in Müller glia also failed to induce neurogenic competence. The authors conclude that although Sox8/9 influence gene expression and nuclear positioning in Müller glia, they are dispensable for retinal gliogenesis.

Overall, the manuscript is well-written and illustrated, with high-quality genetic approaches and multiomic profiling. It provides new insights to the field, particularly by refining prior models regarding the role of SoxE transcription factors in Müller glia specification/maintenance. However, several aspects of the experimental design and data interpretation warrant clarification, and some conclusions appear overstated.

SUGGESTIONS TO AUTHORS

© 2026. Published by The Company of Biologists under the terms of the Creative Commons Attribution License (https://creativecommons.org/licenses/by/4.0/).

Major comments:

1) The timing of Sox8/9 deletion in late-stage retinal precursors raises an important concern regarding the interpretation of these experiments. Müller glia are generated approximately between P2 and P8 (as summarized in Fig. S1A), yet tamoxifen was administered between P3 and P5. Could the authors exclude the possibility that this timing, occurring after the onset of Müller glia specification, is insufficient to impact fate determination? This could explain the divergence from previous studies, such as Muto et al. and Poché et al., which targeted earlier developmental windows. Although the authors acknowledge a timing difference in the Discussion, the conclusion (for instance in the Abstract, line 20) that Sox8/9 are dispensable for Müller cell specification appears overstated. Given the experimental design, the data support conclusions about maintenance of glial identity but probably not about specification.

The reviewer is correct. We have removed all text stating that Sox8/9 are necessary for MG specification. We have modified the Abstract as follows:

"Conversely, conditional deletion of *Sox8* and/or *Sox9* in late-stage progenitors did not impair Müller glia specification, but caused radial displacement of glial nuclei into the outer retina and reduced expression of multiple markers of mature Müller glia. Loss of *Sox8/9* in mature Müller glia modestly increased proliferation post-injury without inducing neurogenic competence. These findings suggest that *Sox8/9* are dispensable for gliogenesis and repression of neurogenic competence, but are essential for proper laminar positioning and full terminal differentiation of retinal Müller glia."

2) The loss of function phenotype is described as "displacement of MG nuclei into the outer nuclear layer." However, in Fig. 2F, while displacement is evident, it is rarely into the ONL itself. The description should more accurately reflect the observed phenotype (for instance "displacement toward the outer nuclear layer").

This has been corrected, with "into" replaced by "towards" in all relevant cases.

3) In the injury paradigm, the main loss of function phenotype is Müller glia nuclear displacement. This phenotype is not discussed in the context of interkinetic nuclear migration (INM), despite the reported increase in proliferation. Could the observed displacement reflect cell cycle re-entry rather than a defect in laminar positioning per se?

The laminar displacement observed here is indeed consistent with cell proliferation in the NMDA-treated animals. Since we have shown that at least a subset of these cells are proliferating, we have modified the text as follows:

"The presence of small numbers of GFP-positive nuclei in the ONL of NMDA-treated animals is likewise consistent with proliferation-induced interkinetic nuclear migration (Goldman 2014)."

4) Proliferation was assessed in the overexpression condition and in the injury model upon loss of function, but not in the loss of function experiments targeting late-stage retinal progenitor cells. Given the observed nuclear displacement, it would be informative to assess proliferation in this condition as well. This would help establish a potential link between nuclear displacement and proliferation.

Proliferation was assessed in both RaxCreER and GlastCreER models with EdU labeling following tamoxifen induction at P3-5. Pup survival rates were low, which in turn made it very difficult to collect sufficient numbers of animals to assess proliferation in the neonatal period. As a result, we are unfortunately unable to draw any conclusions about whether loss of function of *Sox8/9* leads to increased proliferation in the neonatal period. However, our data does not support the possibility that the observed displacement of MG nuclei represents either active or abortive MG proliferation, since no expression of cell cycle-related genes was observed in *Sox8/9*-deficient MG at any age tested (P17, P60, P90). Even if loss of Sox8/9 in immature MG at P3-5 were to induce abortive cell cycle re-entry, and lead to persistent nuclear displacement, the fact that we observe a substantial increase in the fraction of displaced nuclei from P16 to P90 in

© 2026. Published by The Company of Biologists under the terms of the Creative Commons Attribution License (https://creativecommons.org/licenses/by/4.0/).

RaxCreER;*Sox8*$^{lox/lox}$;*Sox9*$^{lox/lox}$;*R26-CAG-lsl-Sun1GFP* animals strongly suggests that this is not the primary cause of the phenotype. We now address this in the text as follows:
"This radial displacement resembles the interkinetic nuclear migration seen following injury-induced proliferation in zebrafish (Goldman 2014), and excitotoxic injury does induce limited proliferation in adult MG lacking *Sox8/9*. However, scRNA/snRNA-Seq detects no cell cycle gene expression in *Sox8/9*-deficient cells at any age that we profiled, indicating that displaced cells are not proliferating. Although displacement may partly reflect abortive cell cycle re-entry in immature MG, the progressive increase in displaced nuclei from P16 to P90 in *RaxCreER*;*Sox8*$^{lox/lox}$;*Sox9*$^{lox/lox}$ animals (Fig. 3D) (Fig. 3D) argues that this is not the primary cause of the observed phenotype."

5) Along the same line, the authors highlight Sox8/9 target genes associated with cell migration. However, if Müller glia nuclear displacement is related to INM, one might expect enrichment of cell cycle-related targets among Sox8/9-regulated genes. Have the authors examined this possibility? Clarifying whether cell cycle regulators are significantly represented in the dataset would strengthen the interpretation.

We do not see altered expression of cell cycle regulators in the list of Sox8/9-regulated genes in Table ST5, suggesting that the radial displacement of mutant MG nuclei may not reflect abortive cell cycle re-entry. This is now addressed explicitly in the text as described for point 4 above.

6) In Fig. 1C, a marked displacement of Rbpms^{+} cells is visible. This phenotype is not discussed although it would deserve explicit commentary to clarify its significance.

This is now discussed as follows:

"Disordered lamination, particularly of Rbpms-positive retinal ganglion cells, was also frequently observed following overexpression of *SOX8* and *SOX9*, either individually or in combination, consistent with disrupted expression of genes controlling cell adhesion and lamination in RPCs (Fig. 1C)."

7) Controls at P16 should be included in Figure 3D and 3I to allow a direct comparison with the control conditions.

Respectfully, we do not believe that these controls are essential for this part of the study. Since we do not observe any displaced cells at P90 in *GlastCreER;Sun1-GFP* mice, and have never observed displaced cells at P14 in previous studies (c.f. de Melo, et al. *J. Neurosci*. 2016), it is very unlikely that we would detect any displaced cells in controls at P16. Previous studies of *RaxCreER;Sun1-GFP* mice induced at P28 and analyzed at P31 did not reveal notable radial displacement of MG nuclei at this age (Pak, et al. PLoS ONE 2014). Because of the high baseline level of nuclear displacement in *RaxCreER;Sun1-GFP* at P90, we de-emphasized use of the *RaxCreER*;*Sox8*$^{lox/lox}$;*Sox9*$^{lox/lox}$ for the remainder of the study, as stated in the text. We nonetheless believe that the P16 data from these animals remains valuable, although we are willing to remove it at the reviewer's request.

8) ANOVA is applied here to small sample sizes (n=3-4 retinas per condition). A non-parametric test such as Kruskal-Wallis would be more appropriate for these sample sizes.

The data has been reanalyzed as suggested.

Minor comments:
1) The reported increase in Mki67+& GFP+/GFP+ cells is not readily apparent in Fig. S2C. Adding arrowheads highlighting double-positive cells would improve clarity.

This has been done.

2) Page 1, lines 41-42: Stolt et al. is cited twice in the same sentence.

© 2026. Published by The Company of Biologists under the terms of the Creative Commons Attribution License (https://creativecommons.org/licenses/by/4.0/).

This has been corrected.

3) Some figures show photoreceptors oriented up, while others show them down.

We have modified the alignment of explant images shown in Fig. 1 and Fig. S2 so that the photoreceptors are consistently positioned towards the top of the image. All other images are already oriented in this position.

Reviewer 3: In their manuscript titled "Sox8 and Sox9 regulate differentiation and nuclear positioning of retinal Müller glia" Pannullo et al. describe the molecular and cellular dissection of the role of key retinal transcription factors in retinal development. They show that Sox8 and Sox9 are expressed in late-stage RPCs and retained in Müller glia from scRNA-seq data. Square wave electroporation of Sox8, Sox9 or the combination had a subtle effect on proliferation and cell fate specification in E14.5 retinal explants as measured by co-localization of GFP and immunofluorescence. Conditional knockout of Sox8, Sox9 or both led to displacement of Müller glial cell nuclei as measured by Sox2 immunoreactivity and other markers. Importantly, there did not appear to be any reactive gliosis that can lead to Müller glial cell displacement. Multiome analysis of purified Sox8/9 deficient Müller glia indicated dramatic changes in gene expression and cut&run showed the landscape of TF binding across the genome. Finally, they deleted Sox8/9 in adult mice and showed there was no increase in neurogenic competence.

1) For the E14.5 electroporation, the scoring of GFP and immunostaining can be challenging in tissue sections. To overcome this limitation and provide more rigorous data on the changes in cell fate with ectopic expression, the manuscript would benefit from dissociated cell staining and scoring. It is possible to visualize the GFP and the immunostaining and determine precise proportions.

We have added scRNA-Seq analysis of FACS-isolated GFP+ cells electroporated with pCAGIG-Sox8/9 at E14 and harvested at P0, as discussed previously. This is now included in Fig. S3 and Table ST1. As discussed, this has led us to somewhat modify our initial conclusions. We confirm a robust reduction in RGC generation, along with induction of late- stage RPC-specific genes – including temporal identity factors such as Nfia and Nfib – in electroporated RPCs. We do not, however, see clear evidence for an increase in numbers of either rod or cone photoreceptors, implying that the observed increases in Otx2 immunoreactivity likely do not reflect induction of later-born cell types. We also observe a remarkable induction of Olig1/Sox10-positive oligodendrocyte lineage cells, which are not observed in controls, and may result from ectopic induction of Sox10, which we identify as a direct target of Sox8/9. The Discussion is expanded to include these new findings, and the discrepancy in Otx2 detection is now covered in the Limitations of the Study section.

2) To independently validate those results in (1) it would also be useful to perform scRNA- seq on the purified cells from the electroporation.

See response to point 1.

3) The omics analysis of the knockout were elegant but it wasn't clear what we learned from those studies. What are the key targets of Sox8/9 in Müller glia? What exactly are the consequences of loss of the TFs? There were no downstream functional studies to validate any of the proposed targets or pathways. It may not be feasible to do more experiments but a discussion would be beneficial.

The following discussion of direct Sox8/9 targets (i.e. genes that are both associated with Sox8/9 CUT&RUN peaks and show altered expression in *Sox8/9* mutants) is included:

"Furthermore, at both P2 and P17, we see that Sox8 and/or Sox9 target sites directly target regulatory sequences associated with Notch pathway genes (*Notch1*, *Hes1/5*), as well as MG-specific genes (*Kcnj10*, *Kdr*, *Fxyd3*) and genes regulating cell migration and adhesion (*Cdh2*, *Ddr1*, *Pcdh10*, *Kit*) that are differentially expressed in *Sox8/9* mutants (Tables ST3 and ST5)."

Please note that we have also added the names of two authors who assisted with analysis of

© 2026. Published by The Company of Biologists under the terms of the Creative Commons Attribution License (https://creativecommons.org/licenses/by/4.0/).

Chx10;Sox9lox/lox mice, and were inadvertently omitted in the initial submission.

We hope that these changes have addressed the Reviewer's outstanding concerns, and look forward to your response.

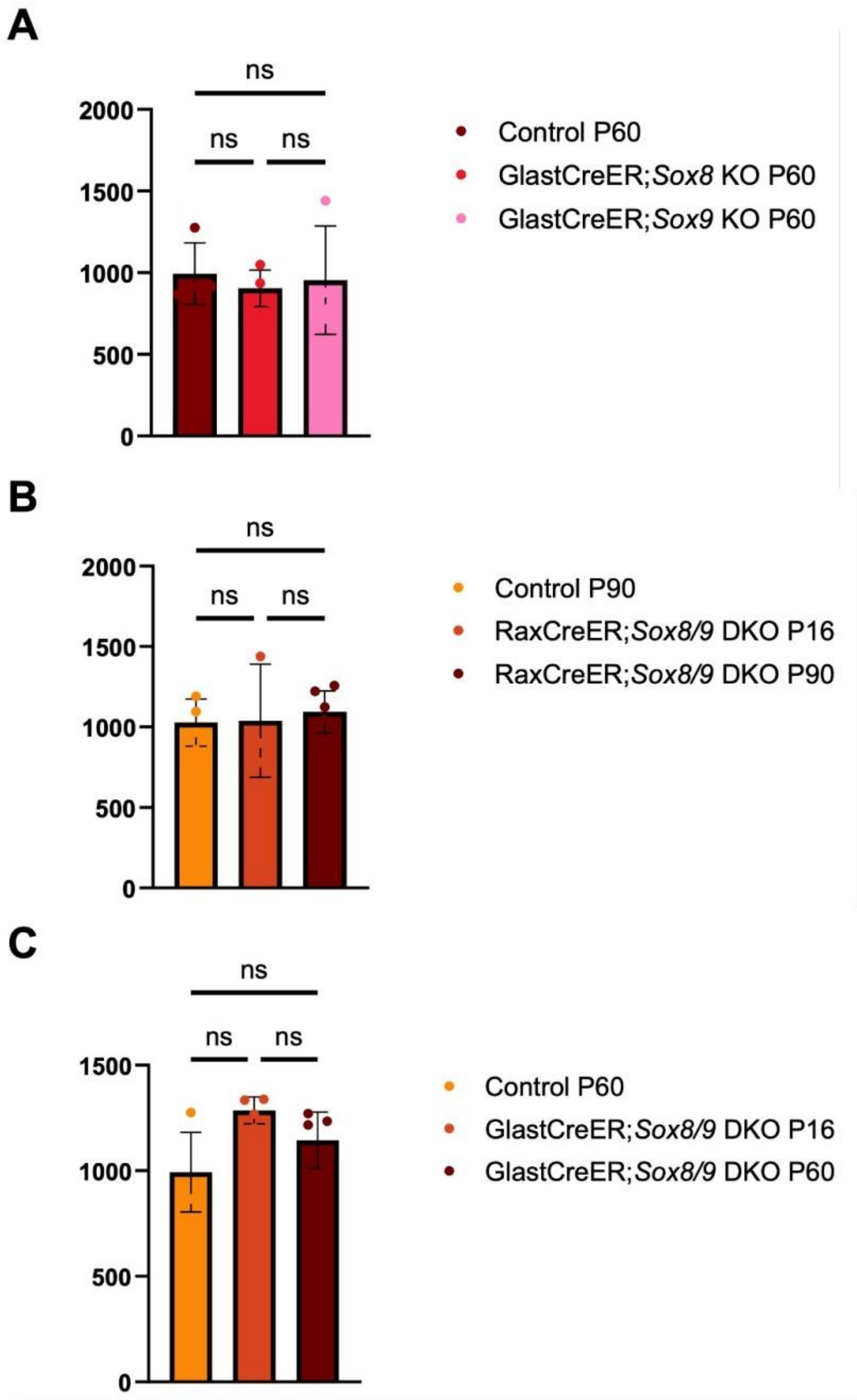

Reviewer Fig. 1
(A-C) Quantification of mean percentage ± SD of Sox2-positive cells in **(A)** control, *GlastCreER;Sox8$^{lox/lox}$;Sun1-GFP*, and *GlastCreER;Sox9$^{lox/lox}$;Sun1-GFP* retinas, **(B)** control and *RaxCreER;Sox8/9$^{lox/lox}$;Sun1-GFP* retinas, and **(C)** control and *GlastCreER;Sox8/9$^{lox/lox}$;Sun1-GFP* retinas. ns = not statistically significant. Significance was determined via Kruskal-Wallis with Dunn's multiple comparisons. Each data point was calculated from an individual retina.

© 2026. Published by The Company of Biologists under the terms of the Creative Commons Attribution License (https://creativecommons.org/licenses/by/4.0/).

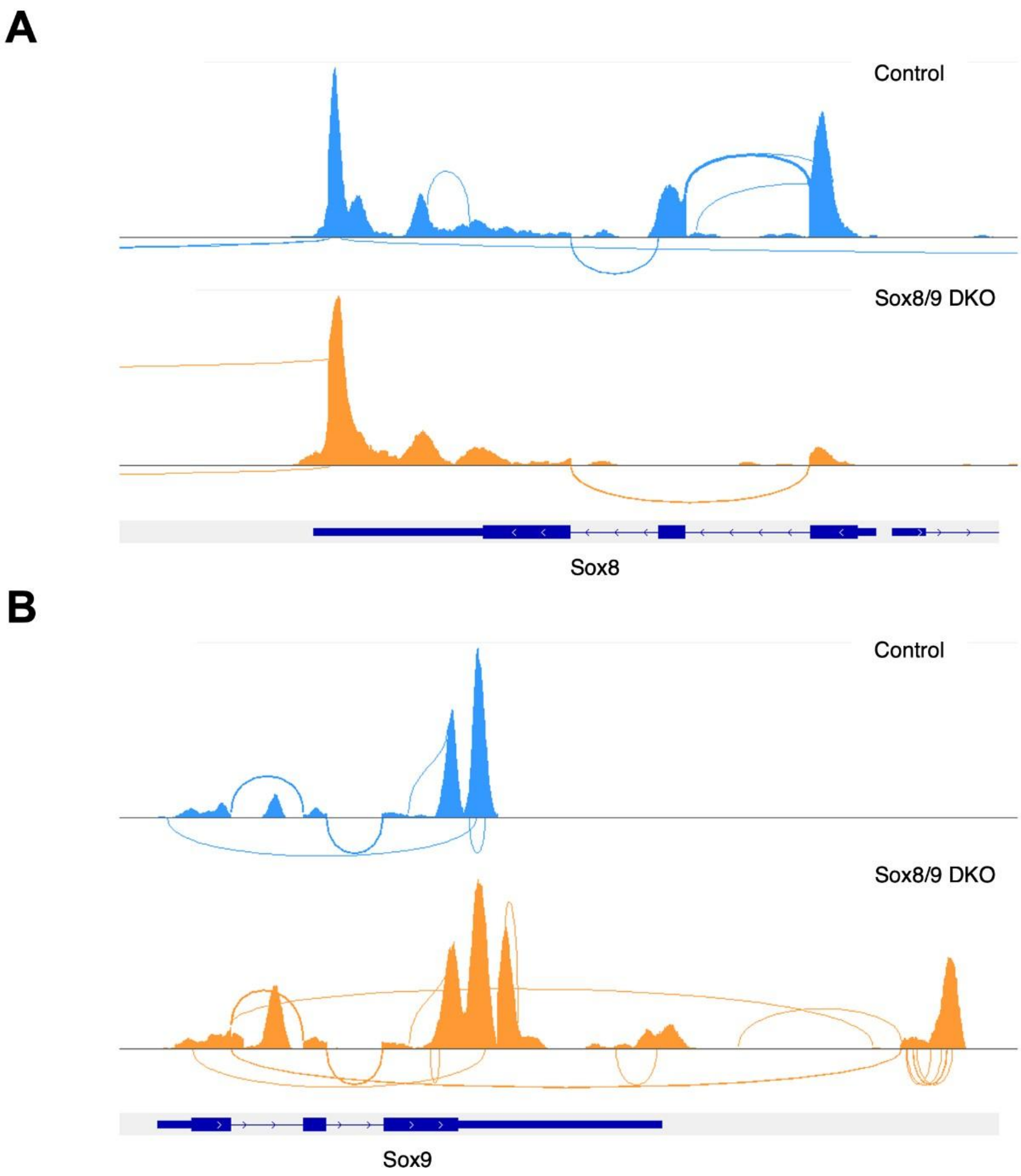

Reviewer Fig. 2
(A-B) Read pile up and sashimi plot for **(A)** *Sox8* and **(B)** *Sox9* in control and *Sox8/9* DKO mature MG.

<u>Second decision letter</u>

MS ID#: dev.205508R1

MS TITLE: Sox8 and Sox9 regulate differentiation and nuclear positioning of retinal Müller glia.

AUTHORS: Nicole Pannullo; Debarpita Datta; Clayton Santiago; Jared Tangeman; Lizhi Jiang; Leighton Duncan; Taqdees Gohar; Megan Gimmen and Seth Blackshaw

Dear Dr Blackshaw,

I am happy to tell you that your manuscript has been accepted for publication in Development, pending our standard publication integrity checks.

© 2026. Published by The Company of Biologists under the terms of the Creative Commons Attribution License (https://creativecommons.org/licenses/by/4.0/).

Please note reviewer 1 raises a point that was not addressed in review. This is not viewed as essential, however in your final version, you can include a discussion of this point or in the limitations section.

Reviewer 1

The authors have added additional data, clarified key points in the text and responded to the comments and suggestions.

One small point raised in the review was not addressed:
For Figure S2C - Not addressed: Why does the combination of Sox8/9 not increase the number of proliferating cells as Sox8 alone does (panel d)?

However, that is a minor point and does not diminish the impact of this study. I have no further concerns.

Reviewer 2

The authors have satisfactorily addressed all my previous comments. I have no further comments and I believe the manuscript is suitable for publication.

Reviewer 3

SUMMARY OF THE ADVANCE MADE IN THIS PAPER AND ITS POTENTIAL SIGNIFICANCE TO THE FIELD

The authors have addressed all of my concerns.

© 2026. Published by The Company of Biologists under the terms of the Creative Commons Attribution License (https://creativecommons.org/licenses/by/4.0/).
